# Abstract papers from the Energy Informatics.Academy Conference 2022 (EI.A 2022)

**DOI:** 10.1186/s42162-022-00219-2

**Published:** 2022-12-21

## Summary of poster papers in the Energy Informatics.Academy Conference 2022

### Zheng Ma^1^, Anna Vocke^2^, Shari Alt^2^, Victoria Schorr^2^, Dirk Werth^2^, Pedro Baptista^3^, Jose Rosado^4,6^, Filipe Caldeira^3,5^, Filipe Cardoso^3,6^, Nur Shakirah Md Salleh^7^, Wahidah Hashim^8^, Henrik Søndergaard^1^, Hamid Reza Shaker^1^, Lasse Kappel Mortensen^1^, Christian T. Veje^9^, Birte Holst Jørgensen^10^, Guangchao Chen^11^, Bo Nørregaard Jørgensen^1^

#### ^1^SDU Center for Energy Informatics, The Maersk Mc-Kinney Moller Institute, University of Southern Denmark, Odense, Denmark; ^2^August-Wilhelm Scheer Institut für digitale Produkte und Prozesse gGmbH, 66123 Saarbrücken, Germany; ^3^Viseu Polytechnic - ESTGV, Viseu, Portugal; ^4^Coimbra Polytechnic - ISEC, Coimbra, Portugal; ^5^CISeD IPV, Viseu, Portugal; ^6^INESC Coimbra, Coimbra, Portugal; ^7^College of Computing and Informatics, Universiti Tenaga Nasional, Kajang, 43000, Malaysia; ^8^College of Computing and Informatics, Universiti Tenaga Nasional, Kajang, 43000, Malaysia; ^9^Department of Mechanical and Electrical Engineering, University of Southern Denmark, Odense, Denmark; ^10^Department of Wind Energy, Society, Market and Policy, Technical University of Denmark, Roskilde Denmark; ^11^College of Materials Sciences and Opto-Electronic Technology, University of Chinese academy of sciences, Beijing, China

##### **Correspondence:** Zheng Ma (zma@mmmi.sdu.dk)

*Energy Informatics* 2022, 5(3)

The Energy Informatics.Academy Conference 2022 (EI.A 2022) [1] has collected great contributions from researchers and practitioners in various scientific, technological, engineering and social fields to disseminate original research on the application of digital technology and information management theory and practice to facilitate the global transition towards sustainable and resilient energy systems.

With the whole technical program committee’s effort, in total thirty-two (32) high-quality papers (including full papers and short papers) are accepted and presented at the conference. Among the thirty-two papers, five poster papers cover four aspects of the energy informatics domain (shown in Table 1).

Table 1 Themes of the five abstracts from Energy Informatics.Academy Conference 2022 (EI.A 2022)

**Table:** 

Theme	Paper title
Software and applications in energy	Design of an intelligent trading platform for flexibility potentials of private households in the low-voltage grid
	Design of Data Management Service Platform for Intelligent Electric Vehicle Charging Controller—Multi-charger Model
Big data and AI in energy	Long Short-Term Memory on Electricity Load Forecasting: Comparison of Feature Scaling Techniques
Simulation and modeling in energy	Automatic Process Monitoring in a District Heating Substation Utilizing a Contextual Shewhart Chart
Energy informatics projects and analysis	A probabilistic approach to reliability analysis of district heating networks incorporation censoring: A report of implementation experiences

The paper (Title: Design of an intelligent trading platform for flexibility potentials of private households in the low-voltage grid) presents the design of a low-threshold energy market for the intelligent trade of small flexibility potentials between grid operators and private households. The market design and the corresponding trading platform have been developed within the FlexChain project. The platform combines traditional IT infrastructure with blockchain elements to ensure economically balanced trading of the low-price flexibility potentials.

The paper (Title: Design of Data Management Service Platform for Intelligent Electric Vehicle Charging Controller—Multi-charger Model) introduces a multi-charger architecture suitable for single-use and shared Electric Vehicle Charging connection points. This multi-charger architecture allows using one or several common charging points by applying a mesh network of intelligent chargers orchestrated by a residential gateway. It can manage the generated data load and enable the data flow between several independent data producers and consumers.

The paper (Title: Long Short-Term Memory on Electricity Load Forecasting: Comparison of Feature Scaling Techniques) applies the Long Short-Term Memory machine learning algorithm with historical electricity load data from a residential area in Denmark to predict the current electricity load demand. It was found that the Robust Scaler scored the highest R-squared value between 0.90 and 0.95. The R-squared value of the Power Transformer Scaler was between 0.89 and 0.92, whereas the R-squared value of the MinMax Scaler was 0.85.

The paper (Title: Automatic Process Monitoring in a District Heating Substation Utilizing a Contextual Shewhart Chart) presents a data-driven process monitoring methodology called contextual Shewhart chart that is a modified version of the Shewhart chart with a district heating substation data in Denmark. The result shows that the shifts of a process variable’s normal operating range can not be captured by a regular Shewhart chart, but the proposed contextual Shewhart chart by using a contextual variable to vary the acceptable operational ranges based on the identified external factor.

The paper (Title: A probabilistic approach to reliability analysis of district heating networks incorporation censoring: A report of implementation experiences) employs a probabilistic proportional hazard modeling approach to district heating pipe reliability analysis. The approach can model the time-dependent survival probability of pipe assets as a function of asset-related and environmental predictors, which have been shown to influence failure probability in previous studies. However, the result shows that the application of this modeling approach in district heating is challenged by several issues pertaining to data.

**List of abbreviations**

- EI.A: Energy Informatics.Academy

**References**

1. EnergyInformatics.Academy. Energy Informatics.Academy Conference 2022 (EI.A 2022), Vejle Denmark, 24–25 August 2022. https://www.energyinformatics.academy/eia-2022-conference, Accessed 08 September 2022

## Design of an intelligent trading platform for flexibility potentials of private households in the low-voltage grid

### Anna Vocke^1^, Shari Alt^1^, Victoria Schorr^1^, Dirk Werth^1^

#### ^1^August-Wilhelm Scheer Institut für digitale Produkte und Prozesse gGmbH, 66123 Saarbrücken, Germany

##### **Correspondence:** Anna Vocke (anna.vocke@aws-institut.de)

*Energy Informatics* 2022, 5(3)

**Summary:** With an increasing number of renewable energy sources entering the energy mix, the demand for novel, smart stabilization methods for the volatile electricity grid is as pressing as ever. In this paper, we present the design of a low-threshold energy market for the intelligent trade of small flexibility potentials between grid operators and private households. The market design and the corresponding trading platform have been developed within the FlexChain project. The platform combines traditional IT-infrastructure with blockchain elements to ensure economically balanced trading of the low-price flexibility potentials. As a key element of the trading platform, the blockchain technology provides verification of and trust in trading results. The developed design will be implemented and field-tested in the upcoming project phases and economically evaluated with regard to all stakeholders. In this evaluation, special focus will be placed on the different options of blockchain integration.

**Keywords:** Smart Grid, Flexibility, Energy Market, Home Energy Management System, Blockchain

INTRODUCTION

The fluctuating energy production from renewable energy sources makes the power supply in electricity grids increasingly volatile. The risk for periods of grid congestion is furthermore increased by a rising number of large electric consumers finding their way into private households, such as electric vehicles or heat pumps. However, these novel technologies not only pose problems for the future energy grid but bear large potentials for grid stabilization measures themselves. Electric vehicles, private photovoltaic systems and battery storages are flexibility assets, which can be activated given an economic stimulus for the household.

The research project “Blockchain-induced activation of small flexibilities in the low-voltage grid (FlexChain)” [1] is funded by the German Federal Ministry for Economic Affairs and Energy (BMWi) and runs under the funding code 03EI6036A. Its research goal is the development and in-field testing of a low-threshold energy market, providing distribution system operators (DSOs) a platform to incentivize the grid-serving activation of small flexibility potentials in private households. In this paper, we report findings from the development and planned in-field testing of the low-threshold energy market and introduce its architecture, which has been developed within the first project phase.

A key challenge in the trade of small flexibility potentials is the creation of an economically balanced market, since the low traded value of micro flexibility runs the risk of being exceeding by the corresponding transaction costs. The performance of the presented market design for all stakeholders as well as further research questions will be evaluated in an in-field testing starting in the beginning of 2023. The acquired data will, among others, be used to evaluate the economic advantages of a blockchain-based trading environment over traditional, centralized technologies.

RELATED WORK

In addition to the traditional stabilization of the electricity grid by large, centralized generators [2], the possibility of stabilizing the grid by demand-side resources [3] is receiving more and more attention in research. These potential resources include households, which can be either traditional consumers or simultaneous electricity consumers and producers, so-called prosumers. Households can be motivated to show grid stabilizing behavior on transmission grid level and distribution grid level [4]. A wide range of appliances are suitable for providing flexibility assets in a household. In this context, different types of household appliances can be distinguished. A first differentiation can be made between appliances that are constantly controlled by a thermostat, such as water heaters and heat pumps, and those that are started and stopped manually [5]. These include, for example, washing machines and dishwashers. The flexibility potential of the latter varies strongly throughout the day, as they are not operated constantly and are therefore subject to uncertainties. However, their general flexibility potential has been demonstrated [6]. A high potential is acknowledged to the supply of flexibility by private electric cars and battery storages [7]. In general, the versatile flexibility potentials of households have been discussed and presented extensively [8–10]. To be able to control and determine the residential flexibility potentials in a targeted manner, the advantages of the use of Home Energy Management Systems (HEMS) have been pointed out [4, 11].

Different goals can be pursued with the activation of residential flexibility potentials. These range from optimizing private electricity generation and consumption of renewable energy sources to grid-serving applications. The latter is an important component of stabilization measures in future grid operation, especially in the context of the energy transition. In Germany, several government-funded research projects such as the Altdorfer Flexmarkt [12] or Flex4Energy [13] are already working on activating the flexibility potential of households for targeted grid stabilization. These projects differ primarily in terms of the devices considered for flexibility provision and the trading mechanism [14]. All related projects share the challenge of the economic efficiency of such energy trading. Economic trading requires transaction costs not to exceed the economic value of the traded flexibility. Since residential flexibility potentials are small, their economic value is low. Novel technologies such as blockchains are seen as a new possibility to master the economic challenge. Currently, German research projects such as pebbles [15] and European collaborative projects such as BRIGHT and eDREAM are already investigating the use of blockchain technology in the context of peer-2-peer energy trading [16]. In contrast to FlexChain’s focus of the grid-serving use of flexibility, these projects focus on increasing the rate of self-consumption of renewable energy or increasing the degree of autarky in local micro grids. In fully mapping the complex market mechanism with blockchain technology, the referenced peer-2-peer projects are challenged by the economic viability of the trading [16]. It is therefore necessary to investigate whether the targeted use of blockchain technology in a minimally designed trading system can reduce transaction costs compared to traditional technologies and thus ensure economically balanced trading in micro-flexibility markets.

THE FLEXCHAIN APPROACH

The goal of the research project FlexChain is the activation of micro prosumer flexibility for grid-serving purposes. Thereby, conventional grid stabilization measures such as cost-intensive and time-consuming infrastructure measures can be avoided and grid expansion can be kept at a minimum. Through the use of flexibility potentials in the distribution grid, the overall cost saving potential amounts to up to 55% by 2035 [17]. The general idea of FlexChain is to allow the grid operator to purchase the flexibility available in participating households in a trade. If households reduce or increase their electric power consumption in accordance with the traded amount and activation time, the grid operator can use this to resolve bottlenecks in the grid. A prerequisite is the forecasting and activation of private flexibility potentials on the one hand, and on the other hand the capability of the grid operator to predict grid bottlenecks and determine its flexibility demand based on this. A schematic of the FlexChain approach is shown in Figure 1. The matching of the available flexibility potentials of the households and the flexibility required by the grid operator takes place in a trading environment with blockchain elements. The blockchain modules are intended to contribute to the economic efficiency, security and confidentiality of the trading system. Data exchange between the household and the trading platform as well as between the network operator and the trading platform must be compatible with the German standard for secure data communication via the Smart-Meter-Gateway (SMGW).

This described approach leads to the following research questions, which need to be answered for the practical implementation of the developed flexibility market:

- Which household appliances are used as flexibility assets?
- How is the price of a flexibility determined?
- In which time frame does the trading take place?
- What is the size of a traded flexibility?
- How can trading be structured in a goal-oriented manner using blockchain technology?

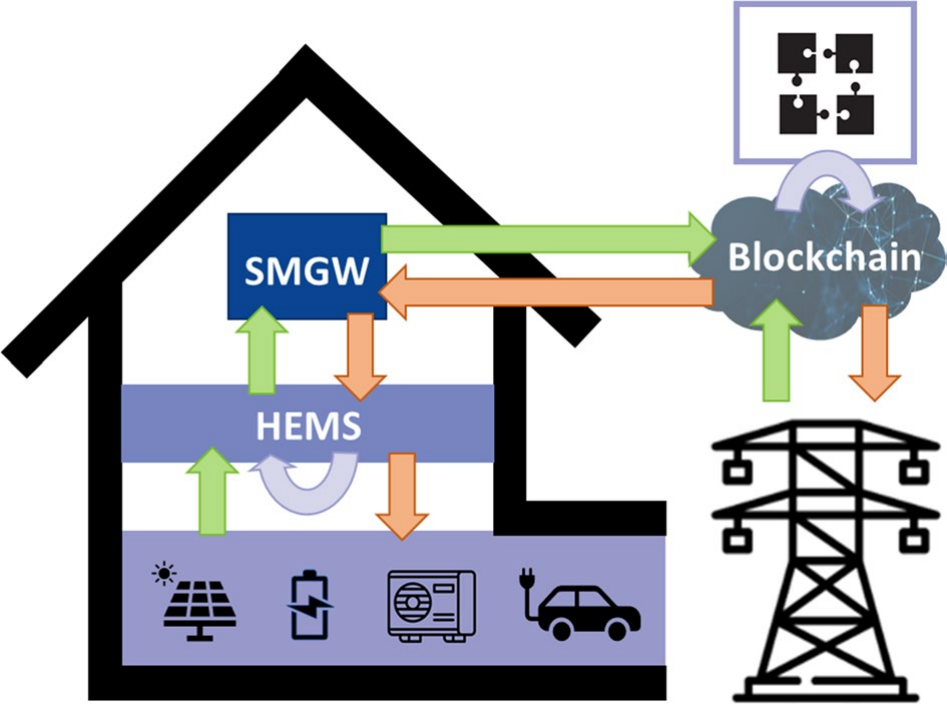


Figure 1 Schematic of the FlexChain approach

DESIGN OF THE FLEXIBILITY MARKET

FlexChain’s first project phase has been dedicated to the analysis and determination of the requirements to the energy trading platform. In this phase, the project partners evaluated and specified the participants of the energy market together with their obligations and rights, potential flexibility assets, adequate market types, trading time frames and the integration of blockchain technology in the market’s IT-infrastructure. The result of this project phase is the market design, which is presented in this section and will be implemented and field-tested in the upcoming project phases.

Figure 2 displays a schematic of the market design. Two types of roles participate in the energy trade: the flexibility provider, i.e. the private household represented by its HEMS, and the DSO. The flexibility provider is the end consumer in the low-voltage electricity grid and, in the case of an installed private photovoltaic system, a prosumer. The role of the second market participant, the DSO, is manifold: He acts as an administrator of the market platform and the implemented blockchain and is responsible for the grid simulation and forecasting of grid congestion intervals. As these obligations exceed the standard purview of a DSO, the tasks are either outsourced to capable project partners or third-party providers in FlexChain.
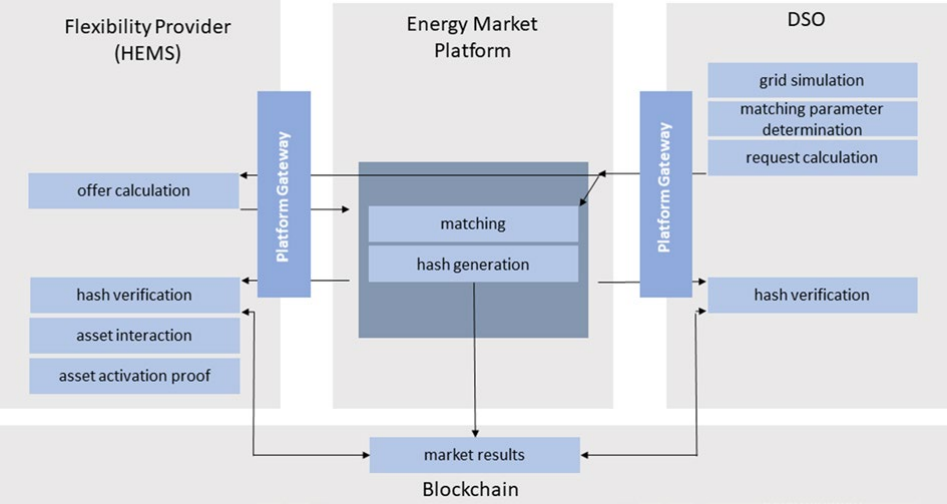


Figure 2 Schematic of the market design

A flexibility trade follows a defined sequence. It is initiated by the DSO, who sends a flexibility request, calculated based on grid simulations, to the platform environment. The grid simulation result includes information on households which are, based on their location in the electricity grid, suitable for resolving the potential congestion event. It furthermore holds details on the requested flexibility, i.e. the corresponding time slot and amount of flexibility, as well as market parameters adjustable by the DSO, which are the market type (fixed price or auction), the offered price and the minimum percentage of the requested flexibility that must be met by valid flexibility offers for the trade to be processed. The traded power and time units of the energy market are set to 0.5 kW and 15 min, respectively, corresponding to traded energy blocks with a size of 125 Wh.

Requests opened by the DSO are transferred to all suitable HEMS via the platform. When a HEMS receives a flexibility request, it internally calculates the flexibility potential of the flexibility assets connect to it and, if a matching flexibility potential is found, returns an offer to the central platform. The extension of existing HEMS software by the required functionalities, i.e. the forecasting of flexibility potentials as well as internal offer calculation, is one of the project’s research goals.

On the market platform, a matching algorithm, which was developed within the project, is run to select offers fitting a given flexibility request. The algorithm allows for a variety of offer selection modes such as first-come-first-served or minimal-number-of-required-contracts in the case of a fixed-price market or lowest-bid selection in an auction market. The performance of the different selection modes for all stakeholders will be evaluated within the project. The achieved market result is communicated with the DSO and all bidding HEMS, which consequently activate their traded flexibility potential.

After a successful matching of a flexibility request and flexibility offers, a Merkel tree [18, 19] for the achieved market result is calculated on the market platform and passed to a dedicated blockchain. Using this Merkel tree imprinted on the blockchain, the market result can be verified through hashes generated with the market results received by the HEMS and the DSO. In this way, the consistency of the distributed market outcome can be confirmed. This mechanism is intended to increase user trust in the developed energy market. Economic aspects of a market concept, in which the entire market processing including offer matching, contract generation and contract execution is implemented on a blockchain e.g. via smart contracts, has been evaluated in a preliminary analysis. These first investigations could not imperatively show the economic benefit of an exclusive use of blockchain technology, in which traded returns must balance transaction costs. The project consortium thus agreed on developing an agile, modular software and hardware environment connected to a blockchain element. This infrastructure will be used to compare the financial balance of a non-blockchain, partly-blockchain and full-blockchain approach and, based on the result of this analysis, can be easily transformed into a full-blockchain setup.

Following a successful matching of flexibility requests and offers, participating HEMS are obliged to activate the traded flexibility potential and provide proof of the activation. In return, the DSO must initiate the processing of the traded refund. The content and specification of the required activation proof is subject of current research activities.

To be able to react to short-term congestion in the distribution grid, the presented market follows an intra-day trading, which is characterized by a fixed timeline for the sending of flexibility requests, offer placing, the trading period of a certain flexibility, the corresponding activation of the flexibility as well as the final confirmation of this activation. Since trading takes place within a defined time slot, which corresponds to the standard duration of 15 min, the computational effort and thus the transaction costs can be kept at a minimum.

In FlexChain, residential flexibility potentials are generated by private heat pumps, battery storages, charging stations for electric vehicles and photovoltaic systems. These flexibility assets are controllable via the HEMS, whose development is part of the project’s research aims, and can be managed with no or minor impact on user comfort. An integration of comfort-related household devices such as washing machines, kitchen devices or entertainment technologies lowers participant acceptance and prevents device management without active residential participation. In order to provide a low-threshold entry into the energy market and guarantee its easy-to-use design, automated background administration of flexibility assets is set as a goal.

OUTLOOK

The presented energy market as well as accompanying research questions will be evaluated in a field test. Via economic incentives, e.g. reduced rates on the installation of a compatible battery storage system, five to ten prosumers in the low-voltage grid administered by the Stadtwerke Saarlouis, a mid-sized DSO in FlexChain’s research consortium, are invited to participate. A prerequisite for participation is a functioning private photovoltaic system at the start of the field test. Since the number of participants does not suffice neither to generate nor to solve a congestion event, synthetic grid congestion will be simulated via a scaling factor for the grid capacity.

The in-field testing serves the purpose of scrutinizing the entire IT-infrastructure, the interplay of the different software modules and the fine tuning of all adjustable market parameters, such as time intervals defining the trading process, in a real-life application. The main research questions to be answered concern the economic aspects of the presented energy market, e.g. to which extend a blockchain-based market economically outperforms traditional technologies or whether smart energy trading is an economic alternative to grid expansion.

To answer these questions holistically, in-field data is needed, which intrinsically respects non-simulatable aspects such as unpredicted user behavior.

**List of abbreviations**

- DSO: Distribution System Operator
- HEMS: Home Energy Management System
- SMGW: Smart-Meter-Gateway

**Acknowledgements:** This work is part of the research project “Blockchain-induced activation of small flexibility potentials in the low-voltage grid (FlexChain)” funded by the BMWi. The funding code is 03EI6036A. In addition to the August-Wilhelm Scheer Institut für digitale Produkte und Prozesse gGmbH (consortium leader), the project partners are Hager Electro GmbH & Co. KG, VIVAVIS AG, Oli Systems GmbH, and Stadtwerke Saarlouis GmbH. The project has a three-year duration and is estimated to end in mid-2023.

**References**

1. Torabi-Goudarzi, S, Werth D, Alt S (2021) Abstracts from the Energy Informatics.academy asia 2021 conference and Phd workshop. Energy Informatics 4:9–11
2. Lannoye E, Flynn D, O'Malley M (2012) Evaluation of power system flexibility. IEEE Transactions on Power Systems 27:922–931
3. Aghaei J, Alizadeh M-I (2013) Demand response in smart electricity grids equipped with renewable energy sources: A Review. Renewable and Sustainable Energy Reviews 18:64–72
4. Khajeh H, Laaksonen H, Gazafroudi AS, Shafie-khah M (2019) Towards flexibility trading at TSO-DSO-Customer Levels: A Review. Energies 13:165
5. Amayri M, Silva CS, Pombeiro H, Ploix S (2022) Flexibility characterization of residential electricity consumption: A machine learning approach. Sustainable Energy, Grids and Networks 32:100801
6. D’hulst R, Labeeuw W, Beusen B, Claessens S, Deconinck G, Vanthournout K (2015) Demand response flexibility and flexibility potential of residential smart appliances: Experiences from large pilot test in Belgium. Applied Energy 155:79–90
7. Ma Z, Jørgensen BN (2018) A discussion of building automation and stakeholder engagement for the readiness of Energy Flexible Buildings. Energy Informatics. https://doi.org/10.1186/s42162-018-0061-z
8. Jensen SØ, Marszal-Pomianowska A, Lollini R, Pasut W, Knotzer A, Engelmann P, Stafford A, Reynders G (2017) IEA EBC Annex 67 energy flexible buildings. Energy and Buildings 155:25–34
9. Stinner S, Huchtemann K, Müller D (2016) Quantifying the operational flexibility of building energy systems with thermal energy storages. Applied Energy 181:140–154
10. Wanapinit N, Thomsen J, Kost C, Weidlich A (2021) An MILP model for evaluating the optimal operation and flexibility potential of end-users. Applied Energy 282:116183
11. Pinto R, Bessa RJ, Matos MA (2017) Multi-period flexibility forecast for low voltage prosumers. Energy 141:2251–2263
12. Koepple S, Lang C, Bogensperger A, Estermann T (2019) Altdorfer Flexmarkt—Decentral flexibility for distribution networks. International ETG-Congress 2019; ETG Symposium: 1–6
13. Straub S (2018) Flex4Energy—Flexibilitätsmanagement für die Energieversorgung der Zukunft: Abschlussbericht: Laufzeit des Vorhabens: 01.04.2015–31.03.2018, ads-tec GmbH, Nürtingen
14. Torabi-Goudarzi S, Alt S (2021) Research on activation of flexibility potentials in a market-oriented process. 2021 5th International Conference on Smart Grid and Smart Cities (ICSGSC). https://doi.org/10.1109/icsgsc52434.2021.9490501
15. Peer-to-peer-energiehandelauf basis von blockchains. In: Pebbles Projekt. https://pebbles-projekt.de/. Accessed 27 Jul 2022
16. Antal C, Cioara T, Antal M, et al. (2021) Blockchain based decentralized local energy flexibility market. Energy Reports 7:5269–5288
17. Regulatorischer handlungsbedarf zur Erschließung und Nutzung …—Dena. https://www.dena.de/fileadmin/dena/Publikationen/PDFs/2019/Dena-ANALYSE_Regulatorischer_Handlungsbedarf_zur_Erschliessung_und_Nutzung_netzdienlicher_Flexibilitaet.pdf. Accessed 27 Jul 2022
18. Merkle RC (1988) A digital signature based on a conventional encryption function. Advances in Cryptology—CRYPTO’87 369–378
19. Merkle R (1982)

## Long short-term memory on electricity load forecasting: comparison of feature scaling techniques

### Nur Shakirah Md Salleh^1^, Bo Nørregaard Jørgensen^2^, Wahidah Hashim^3^

#### ^1^College of Computing and Informatics, Universiti Tenaga Nasional, Kajang, 43000, Malaysia; ^2^SDU Center for Energy Informatics, Mærsk Mc-Kinney Møller Institute, University of Southern Denmark, Odense, 5230, Denmark; ^3^College of Computing and Informatics, Universiti Tenaga Nasional, Kajang, 43000, Malaysia

##### **Correspondence:** Nur Shakirah Md Salleh (shakirah@uniten.edu.my)

*Energy Informatics* 2022, 5(3)

**Summary:** Electricity load prediction can assist utility companies to estimate the electric power to be generated. The study used historical electricity load data from a residential area in Denmark, and employed a single feature, i.e., the previous hour’s electricity load, to predict the current electricity load demand. Due to the different data ranges found in the dataset, this manuscript intended to prove the importance of feature scaling technique selection that would impact the prediction results. A comparison was made on the prediction results of the scaled dataset using the MinMax Scaler, Robust Scaler, and Power Transformer Scaler. The machine learning algorithm, Long Short-Term Memory (LSTM), was applied because the input and output data were in time series, and the estimation of electricity load value was the expected output. It was found that the Robust Scaler scored the highest R-squared value between 0.90 and 0.95. The R-squared value of the Power Transformer Scaler was between 0.89 and 0.92, whereas the R-squared value of the MinMax Scaler was 0.85.

**Keywords:** LSTM, Electricity Load, Robust Scaler, Power Transformer Scaler, MinMax Scaler, R-squared

INTRODUCTION

In certain countries such as the United States, electricity generation contributes 25% of the primary sources of greenhouse gas emissions [1]. The greenhouse gas emissions impact climate change by trapping heat that leads to global warming. The United Nations (UN) has introduced 17 Sustainable Development Goals (SDGs) that emphasise a holistic approach to achieving sustainable development for all. The seventh SDG goal is “Affordable and Clean Energy”. Renewable energy is currently being used in several countries, such as Denmark, which generates 45% of its electricity from wind [2].

Electricity load forecasting is important for electricity suppliers because it can assist them to generate electricity sufficiently as forecasted. Over generation of electricity incurs additional costs to the electricity suppliers in generating more than the demand, and additional carbon emissions will be produced during electricity generation. Electricity load forecasting happens in time series with the expected value of electricity load. The machine learning algorithms that support time series and regression include Recurrent Neural Network (RNN) and Long Short-Term Memory (LSTM) due to the capability of memorising the information on each iteration. RNN and LSTM can perform training and testing by grouping based on the time steps. However, RNN has limitations in its memory unit. LSTM overcomes the drawback of RNN by providing the option to discard unmeaningful information through the forget gate. LSTM falls under supervised learning, whereby the training is based on the historical electricity load dataset to identify the electricity load pattern. This study focuses on LSTM after the comparison study between LSTM and the feed-forward network, Artificial Neural Network (ANN), showed that LSTM performed better than ANN [3].

Electricity load is represented by kilo Watt per hour (kWh). The electricity load range has a major difference between peak and non-peak hours. The feature scaling technique helps to minimise the impact of one significant number on the model by bringing the various features in the same standing. Many studies have applied MinMax scaler as feature scaling technique to obtain the best model performance in electricity load forecasting. The existing studies often stated the selected feature scaling technique; nevertheless, the studies did not always sufficiently justify the effectiveness of the selected feature scaling technique by comparing it with other feature scaling techniques that may also influence the model efficiency. Other feature scaling techniques that are rarely used in electricity load forecasting are Robust Scaler and Power Transformer Scaler. Therefore, the major objectives of this article are:

- to identify the range of the scaled dataset using the MinMax Scaler, Robust Scaler, and Power Transformer Scaler,
- to compare the evaluation metrics of the model generated by the training dataset scaled using the MinMax Scaler, Robust Scaler, and Power Transformer Scaler, and
- to compare the electricity load between the actual and prediction values generated by the models.

The remainder of the article is organised as follows: the Review of Related Works section discusses similar works that applied the LSTM algorithm, feature scaling techniques applied, and evaluation metric used to evaluate the model. The Methodology section describes the proposal approach for LSTM in this study and its implementation. The Results section displays the implementation results including scaled dataset, model evaluation, and prediction values, followed by the Analysis and Discussion section. Finally, a conclusion is issued in the final section.

REVIEW OF RELATED WORKS

Long Short-Term Memory algorithm

Machine learning is a statistical approach for performing predictions using the model generated from supervised or unsupervised learning. Electricity load forecasting based on historical data is an example of the implementation of supervised learning. The output of electricity load forecasting is a numerical value that represents the prediction result. The most suitable method to predict the continuous values of electricity load forecasting is regression [4]. RNN is employed in time series prediction because it can save the output of a layer and feed it back to the input. It stores the previous input in the internal memory [5–7]. Throughout the learning cycles, the gradient values that carry information become too small and insignificant. LSTM can overcome the vanishing gradients issue in RNN by remembering information for extended periods [7–9]. LSTM works in the form of a chain of repeating modules of a Neural Network (NN) with four interacting layers that communicate with each other [7]. By using RNN and LSTM, the data input transforms from a two-dimensional (2D) array (features and label) into a three-dimensional (3D) array (samples, time step, and feature).

Electricity load forecasting predicts in a sequence of time. Liu et al. [10] applied RNN to perform electricity usage prediction. The research by Hossen et al. [11] showed that LSTM performed better than RNN. Previous research by Lee and Choi [12], Zheng et al. [13], and Hossen et al. [11] compared LSTM with Gated Recurrent Unit (GRU). LSTM still demonstrated the best result among other algorithms. Kumari et al. [14] implemented deep learning by applying LSTM, and it produced a low error value for the model evaluation.

Feature scaling techniques

Feature scaling technique, also known as scaler, shifts and rescales values into a certain range without alerting the distribution values [15]. It is used to scale the training data to achieve non-dimensionality and accelerate convergence. Feature scaler helps to minimise the gap in dataset values. A series of article reviews were made to identify the common feature scaling techniques used by other researchers. Nevertheless, not all articles related shared the same scaler techniques applied in their studies.

Table 1 Summary of feature scaling techniques by other researchers

**Table:** 

Scalers	References
MinMax	Salam and El Hibaoui [8], Wen, Zhou, Yang [16], Fekri et al. [17], Ozer, Efe, Ozbay [18], Peng et al. [19], Somu, Raman, Ramamritham [20], Tang et al. [21], Somu, Raman, Ramamritham [22], Li et al. [23], He, Zheng, Xu [24]
Not specified Rescaled between 0 to 1	Bai et al. [25], Eskandari, Imani, Moghaddam [26], Liu and Lin [27], Jana, Ghosh, Sanyal [28], He et al. [29], Wang et al. [30]
Not specified Rescaled between -1 to 1	Dong, Ma, Fu [31]

Table 1 shows that 10 out of the 17 reviewed articles applied the MinMax scaler in their studies. The MinMax scaler technique transforms the values between zero and one.
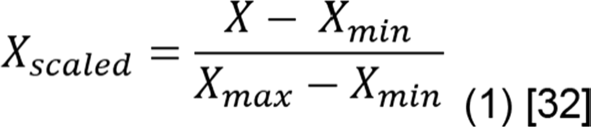


The value X represents the current data to be scaled. X_min_ denotes the minimum value of the dataset feature, while X_max_ signifies the maximum value of the dataset feature. The scaled value of X is generated by the subtraction of the value X and X_min_. Finally, X_scaled_ is divided by the difference between X_max_ and X_min_.

It is challenging to find related works that use Robust Scaler and Power Transformer Scaler in electricity load forecasting. The Robust Scaler minimises the distance between values in the scaled feature by performing the subtraction from the median, and the result is then divided by the interquartile range. This can reduce the importance of outliers [33]. The range of values for the feature is larger than what can be obtained with the MinMax scaler.
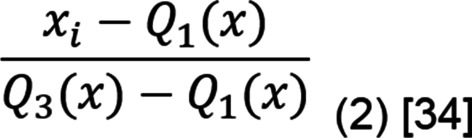


The Power Transformer Scaler transforms input and/or output variables to have a more Gaussian-like distribution. It is believed that this scaler can achieve better performance on a wide range of machine learning algorithms [35]. There are two types of transforms in Power Transformer, namely Yeo-Johnson and Box-Cox.
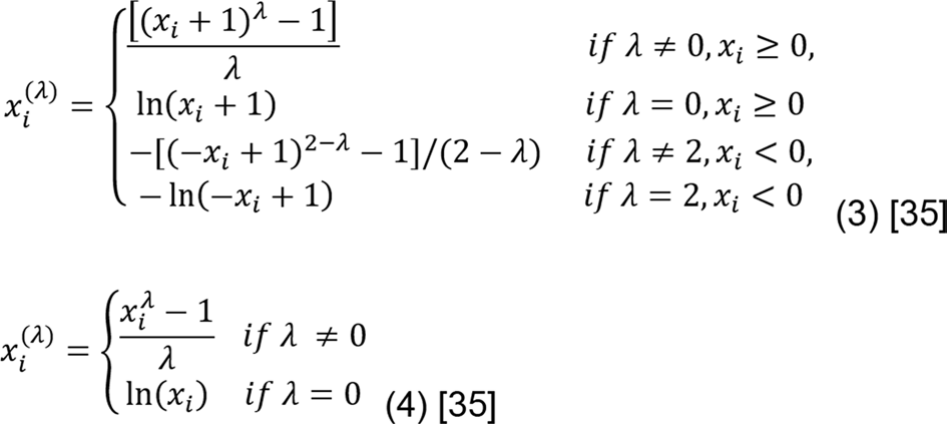


Equation 3 represents the Yeo-Johnson transform that supports positive and negative data, while Equation 4 denotes the Box-Cox transform that only supports positive data [35].

Evaluation metrics

Machine learning requires training and testing phases. The output of the training phase is a model. This model needs an evaluation process to determine its quality. The common evaluation metrics used for regression problems are mean squared error (MSE), root mean squared error (RMSE), mean absolute error (MAE), mean absolute percentage error (MAPE), and R-Squared (R^2^) [33]. MSE evaluates the model by indicating the average of the squares of the error found between the predicted and actual values, while RMSE performs the square root of MSE [33]. MAE indicates the average of the squares of the errors found between the predicted and actual values [33]. For MSE, RMSE, MAE, and MAPE, the model evaluates based on the error values between the test set and the actual value. The error results depend on the dataset range. If the dataset range is between zero and one, the data closer to zero have better results. However, it is challenging to determine the model quality based on the error values if the dataset range is not between zero and one. On the other hand, R^2^ indicates the coefficient of determination of the model by the ratio of the model’s MSE and the difference of the predicted values. R^2^ is the most relevant to regression algorithm because it indicates the quality of the algorithm by capturing the difference of the predicted values [33].

In the related works, the values of evaluation metrics are incomparable because the dataset range is different. For example, RMSE applied in Lee and Choi’s [13] work gave a result of 0.9698. Tongta and Chooruang [36] applied MAE, which provided a result of 36.98. On the other hand, Hossen et al. [12] applied LSTM and used MAPE as an evaluation metric that showed a result between 24 and 35%. Most of the studies were focused on finding the error values instead of finding the variance of the predicted values.

METHODOLOGY

Proposed approach for LSTM

Based on the review made in the previous section, two rarely used feature scaling techniques were considered in this study, which differ from the commonly used scalers in the similar case studies, i.e., MinMax Scaler, and their impact on the output of the selected algorithm, LSTM. The predictions obtained from such pre-processed input led to increased accuracy, as demonstrated by the results obtained. The hourly electricity load prediction was forecast using historical data from the previous hour, with seven days of memory used. The details of the experiments are discussed in detail in this section.

Implementation of LSTM

The dataset used in this study was a residential area in Denmark between 2015 and 2018. The dataset consisted of only one feature: the previous hour electricity load that was represented hourly. The date and time were used as an index of the dataset to be used as a reference to compare the prediction results with the actual electricity load. The dataset used in the training phase was between 2015 and 2017, while the dataset in 2018 was used in the testing phase.
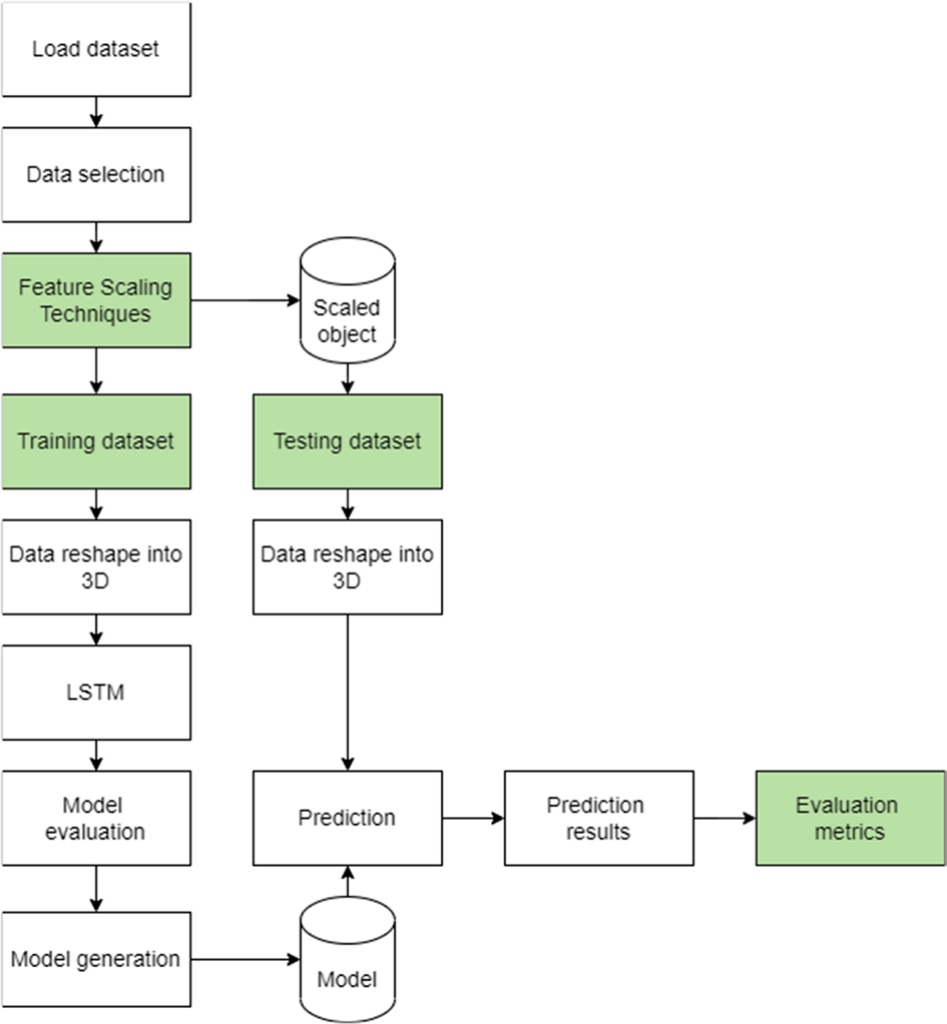


Figure 1 Navigation diagram of the LSTM implementation

Figure 1 shows the navigation diagram of the implementation of LSTM in this study. The highlighted processes are critical in achieving the objectives of this article. The dataset consisted of the date, time, electricity load data of the specific date and time, and the electricity load data of the previous hour. The data selection process selected the feature to be used in the training and testing phases. As mentioned in the previous sub-section, this study only used the previous hour electricity load as the feature. The electricity load data of the specific date and time was used as the label to be used in the training phase. The date and time were assigned as the index of the dataset.

Since the machine learning algorithm used was LSTM, the dataset needed conversion from 2D into a 3D array. The components of the 3D dataset were sample, time steps, and feature. The time steps used in this experiment were 24 and 168 to compare the effectiveness of LSTM in memorising the electricity load pattern every 24 h and 7 days.

LSTM generated a model from the sequential layers of an input layer, hidden layer, and output layer. The hyperparameters for the LSTM layers were set based on Table 2.

Table 2 Hyperparameter used in each layer

**Table:** 

	Input layer	Hidden layer	Output layer
Number of neurons	≈ (Total_training_data * 0.05)	≈ (Total_training_data * 0.05/2)	1
Input shape	Time steps, number of features	None	None
Activation function	Sinc	Sinc	None
Return sequences	True	True	None

Adam Optimiser was used in accelerating algorithm convergence during the training phase with the learning rate applied at 0.001. The Adam optimiser was employed due to its capability adopted from RMSProp, Stochastic Gradient Descent (SGD), and Momentum Optimiser. The characteristics adopted from RMSProp were the abilities to reduce the accumulation gradient in a controlled order and adjust the learning rate accordingly. The Adam Optimiser used one sample at a time, similar to SGD. It adopted the behaviour of the Momentum Optimiser by stabilizing the gradient correction direction.

Another common issue in producing a model is overfitting. This experiment used the epoch value of 100. The early stop mechanism was applied in this study to control the training session so as to not overfit the model.

The model quality from the training session was observed for all three scaled datasets in the model evaluation phase. The loss function that reflected the error between prediction and actual values was used.

Once the loss results showed improvement, the model was ready to be saved and it would be used in the testing phase. The models were named based on the scaler and time steps used. In the testing phase, the testing dataset was used. This testing set was included with an additional 2 weeks of the earlier dataset to warm up the model. The testing dataset was scaled using the scaled object created by the selected scalers. Then, the testing dataset was required to be reshaped into the 3D format to enable it to be used in LSTM. The next process was to perform the prediction of the reshaped data in getting the prediction values. The prediction values were evaluated using the selected evaluation metric, R^2^, to directly compare the prediction and actual values of electricity load hourly.

RESULTS

The comparison of scalers in affecting the model quality was the main objective of this study. The machine learning algorithm used was LSTM, which was expected to produce a more quality model as it could memorise more information. For this reason, the comparison of R^2^ values of 24 time steps and 168 time steps also became a concern in this study. The four months samples were used to represent a peak month in winter (January), spring (April), summer (July), and autumn (October). These four months were selected because they are not in the transition of seasons. In total, there were six models generated in this study: modelR24, modelPT24, modelMM24, modelR168, modelPT168, and modelMM168.

Scaled dataset

The training and testing datasets were scaled to minimise the data gap values. The minimum and maximum values of the data that existed in the dataset are as shown in Table 3.

Table 3 Minimum and maximum value of scaled dataset

**Table:** 

	Robust scaler	Power transformer scaler	MinMax scaler
Minimum	− 1.898697	− 7.349004	0.000000
Maximum	4.122344	3.518321	1.000000

Model evaluation

The first experiment started with the LSTM model generation using 24 h of memory that was represented by time steps. The model evaluation results were derived from the testing dataset that covered the sample of four months, executed monthly. Table 4 shows the evaluation metric of the testing phase dataset scaled using Robust, Power Transformer, and MinMax Scalers.

Table 4 Evaluation metric results of testing dataset for 24 time steps

**Table:** 

Model name	N/A	modelR24	modelPT24	modelMM24
Evaluation metrics	Sample month	Robust scaler	Power transformer scaler	MinMax scaler
MSE	January	124.44	118.45	189.86
	April	37.58	45.33	140.73
	July	17.05	22.86	63.26
	October	41.71	47.39	152.38
MAE	January	7.82	7.31	10.09
	April	4.69	5.05	9.10
	July	3.13	3.58	6.18
	October	4.91	5.07	9.64
RMSE	January	11.16	10.88	13.78
	April	6.13	6.73	11.86
	July	4.13	4.78	7.95
	October	6.46	6.88	12.34
R^2^	January	0.88	0.88	0.81
	April	0.93	0.92	0.76
	July	0.94	0.92	0.78
	October	0.93	0.92	0.73

The second experiment commenced with the LSTM model generation using 168 h of memory information represented by time steps. Table 5 displays the evaluation metric of the testing phase dataset scaled using Robust, Power Transformer, and MinMax Scalers.

Table 5 Evaluation metric results of testing dataset for 168 time steps

**Table:** 

Model name	N/A	modelR168	modelPT168	modelMM168
Evaluation metrics	Sample month	Robust scaler	Power transformer scaler	MinMax scaler
MSE	January	49.78	83.51	161.89
	April	41.25	56.83	87.47
	July	26.13	30.82	40.71
	October	31.98	50.23	100.38
MAE	January	5.24	6.54	9.65
	April	4.76	5.50	7.62
	July	3.98	4.24	5.03
	October	4.22	5.14	8.00
RMSE	January	7.06	9.14	12.72
	April	6.42	7.54	9.35
	July	5.11	5.55	6.38
	October	5.66	7.09	10.02
R^2^	January	0.95	0.92	0.84
	April	0.93	0.90	0.85
	July	0.90	0.89	0.85
	October	0.95	0.92	0.85

Prediction results

Figure 2 represents the prediction results by the model generated using 168 time steps. The results were shown in kWh. The actual electricity load was labelled in red. The observation was made based on the similarity of the prediction values by each model with the actual data.
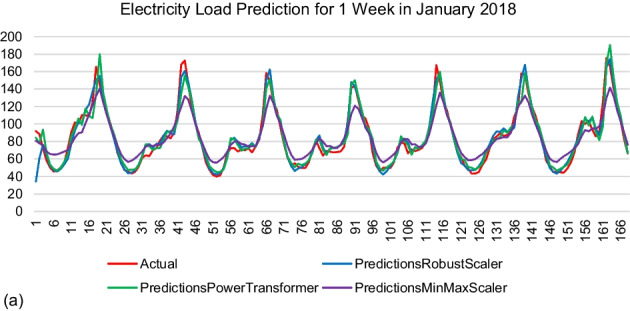



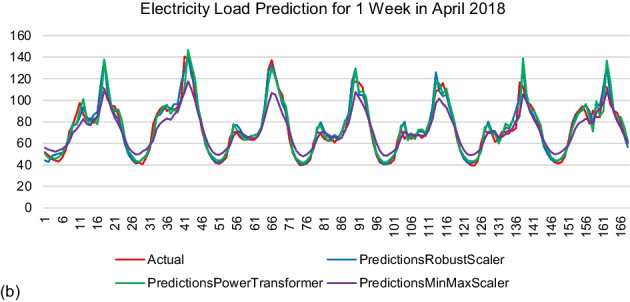



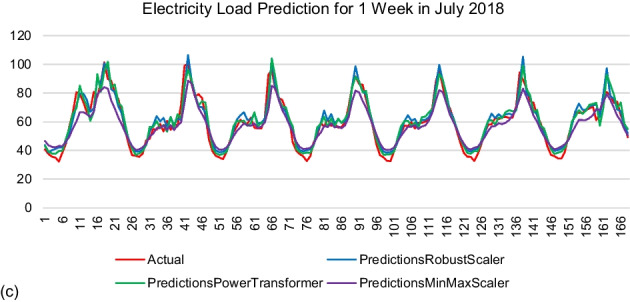



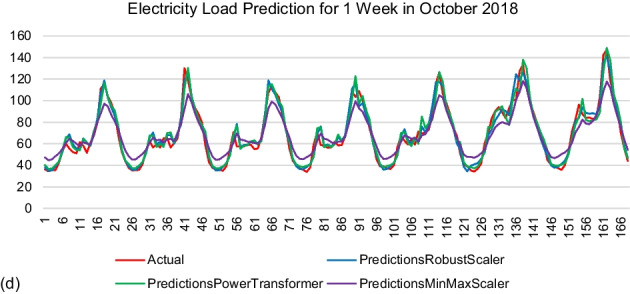


Figure 2 Prediction results by modelR168, modelPT168, and modelMM168. (a) Winter (b) Spring (c) Summer (d) Autumn

ANALYSIS AND DISCUSSIONS

The MinMax scaler only limited the data range to be scaled between zero and one. This resulted in the scaled value becoming too small. The Robust Scaler scaled the dataset between negative one and five, while the Power Transformer Scaler scaled the dataset between negative seven and three. This range was double as compared to the Robust Scaler.

As mentioned from the beginning, the result of the model evaluation metric focused on R^2^ since it compared actual and predicted values. The MinMax Scaler performed the lowest R^2^ value as compared to the other scalers for the 24 time steps and 168 time steps. The R^2^ value between the Robust Scaler and Power Transformer Scaler was very competitive for each sample month for both time steps. The highest R^2^ value was scored by the Robust Scaler when the time steps were set to 168, which represented one week of memory information. The LSTM model could learn better when it was fed with sufficient information through the 168 time steps applied. However, the MinMax scaler failed to reach the peak and valley values of all prediction samples.

CONCLUSION AND FUTURE WORKS

The impact of climate change is becoming obvious from day to day, which drives the UN to introduce the 17 SDGs that emphasise a holistic approach to achieving sustainable development for all. This research indirectly supported the seventh SDG goal of “Affordable and Clean Energy” by comparing the mechanisms that could better predict electricity load since electricity generation contributes to the primary source of greenhouse gas emissions. This study intended to compare the various feature scaling techniques and their impact towards the prediction values. The common feature scaling technique used in the reviewed studies was MinMax Scaler. The other feature scaling techniques used for comparison were Robust and Power Transformer Scalers. The Robust Scaler could minimise the outliers, while the Power Transformer Scaler could transform the dataset into a more Gaussian-like distribution. The machine learning algorithm used in this study was LSTM because of its ability in memorising information to solve the regression problem. The details of the implementation of LSTM and the hyperparameters used were mentioned in this article. This study also proved that the ability of LSTM in memorising information for a certain period could improve the model quality. In total, there were six models generated in this study: modelR24, modelPT24, modelMM24, modelR168, modelPT168, and modelMM168. The best model generated was modelR168 with an R^2^ value between 0.90 and 0.95. This model was generated by the training session of 168time steps with the Robust Scaled dataset.

The current study used the Sinc activation function due to the nature of the dataset that had rises and falls in 24 h of electricity load consumption, which looked similar to sine wave; s-shaped, smooth wave that oscillates above and below zero. Future studies should compare the effectiveness by using other activation functions such as rectified linear activation function (ReLU) and tanh hidden layer activation function (tanh). This is another opportunity to explore since this article has proven that the commonly used feature scaling technique, MinMax Scaler, is not always the best scaler to be used.

**List of abbreviations**

- ANN: Artificial Neural Network
- GRU: Gated Recurrent Unit
- LSTM: Long Short-Term Memory
- MSE: Mean squared error
- RMSE: Root mean squared error
- MAE: mean absolute error
- MAPE: Mean absolute percentage error
- NN: Neural Network
- R^2^: R-Squared
- ReLU: Rectified linear activation function
- RNN: Recurrent Neural Network
- SDGs: Sustainable Development Goals
- SGD: Stochastic Gradient Descent
- UN: United Nations

**Acknowledgements:** This work was funded by UNITEN BOLD2021 Fund: J510050002/2021040, Innovation and Research Management Centre (iRMC), Universiti Tenaga Nasional (UNITEN). This paper’s publication was funded by the Sino-Danish Center. The authors would like to thank the Institute of Informatics and Computing in Energy (IICE), UNITEN for providing a platform to collaborate with the Centre for Energy Informatics, Southern Denmark University (SDU).

**References**

1. United States Environmental Protection Agency [Internet]. Washington D.C.: United States Environmental Protection Agency; 2022. Sources of greenhouse gas emissions; [publication date unknown] [cited 2022 Jun 16]. Available from: https://www.epa.gov/ghgemissions/sources-greenhouse-gas-emissions.
2. Energinet [Internet]. Fredericia: Energinet; 2021. Danish electricity generation was greener than ever in 2020; 2021 Jun 22 [cited 2022 Jun 16]. Available from: https://en.energinet.dk/About-our-news/News/2021/06/22/Danish-electricity-generation-was-greener-than-ever-in-2020.
3. Salleh NS, Suliman A, Jørgensen BN. Comparison of electricity load prediction errors between long short-term memory architecture and artificial neural network on smart meter consumer. In: Zaman HB, Smeaton AF, Shih TK, Velastin S, Terutoshi T, Jørgensen BN, Aris H, Ibrahim N, editors. Advances in Visual Informatics: 7th International Visual Informatics Conference (IVIC 2021) (Lecture Notes in Computer Science); 2021 Nov 23–25; Kajang, Malaysia. Switzerland: Springer Cham; 2021. p. 600–9.
4. Dave A. Regression in machine learning [Internet]. Los Angeles: Data Driven Investor; 2018 [cited 2019, Jun 25]. Available from: https://medium.com/datadriveninvestor/regression-in-machine-learning-296caae933ec.
5. Sikiric G, Avdakovic S, Subasi A. Comparison of machine learning methods for electricity demand forecasting in Bosnia and Herzegovina. Southeast Eur. J. Soft Comput. 2013 Nov 24;2(2):12–14.
6. Basile A, Napporn T, editors. Current trends and future developments on (bio-) membranes. Amsterdam: Elsevier; 2020. p. 199–202.
7. Xuan Z, Zhubing F, Liequan L, Junwei Y, Dongmei P. Comparison of four algorithms based on machine learning for cooling load forecasting of large-scale shopping mall. Energy Procedia. 2017 Dec 1;142:1799–1804.
8. Salam A, El Hibaoui A. Comparison of machine learning algorithms for the power consumption prediction:- Case study of Tetouan city. In: El Hibaoui A, editor. 2018 6th International Renewable and Sustainable Energy Conference (IRSEC); 2018 Dec 5–8; Rabat, Morocco. IEEE; 2018. p. 1–5.
9. Memarzadeh G, Keynia F. Short-term electricity load and price forecasting by a new optimal LSTM-NN based prediction algorithm. Electr. Power Syst. Res. 2021 Mar 1;192:106995. Available from: https://doi.org/10.1109/IRSEC.2018.8703007.
10. Liu C, Jin Z, Gu J, Qiu C. Short-term load forecasting using a long short-term memory network. In: IEEE PES Innovative Smart Grid Technologies Conference Europe (ISGT-Europe); 2017 Sep 26–29; Torino, Italy. IEEE; 2017. p. 1–6. Available from: https://doi.org/10.1109/isgteurope.2017.8260110
11. Hossen T, Nair A, Chinnathambi R, Ranganathan P. Residential load forecasting using deep neural networks (DNN). In: 2018 North American Power Symposium (NAPS); 2018 Sep 9–11; Fargo, North Dakota, USA. IEEE; 2018. p. 1–5. Available from: https://doi.org/10.1109/naps.2018.8600549.
12. Lee Y, Choi H. Forecasting building electricity power consumption using deep learning approach. In: 2020 IEEE International Conference on Big Data and Smart Computing (BigComp); 2020 Feb 19–22; Busan, Korea. IEEE; 2020. p. 542–4. Available from: https://doi.org/10.1109/bigcomp48618.2020.000-8.
13. Zheng J, Chen X, Yu K, Gan L, Wang Y, Wang K. Short-term power load forecasting of residential community based on GRU neural network. In: 2018 International Conference on Power System Technology (POWERCON); 2018 Nov 6–8; Guangzhou, China. IEEE; 2018. p. 4862–8. Available from: https://doi.org/10.1109/powercon.2018.8601718.
14. Kumari A, Vekaria D, Gupta R, Tanwar S. Redills: Deep learning-based secure data analytic framework for smart grid systems. In: 2020 IEEE International Conference on Communications Workshops (ICC Workshops); 2020 Jun 7–11; Dublin, Ireland. IEEE; 2020. p. 1–6. Available from: https://doi.org/10.1109/iccworkshops49005.2020.9145448.
15. Esposito D, Esposito F. Introducing machine learning. New York: Pearson Education, Inc. 2020.
16. Wen L, Zhou K, Yang S. Load demand forecasting of residential buildings using a deep learning model. Electr. Power Syst. Res. 2020 Feb 1;179:106073. Available from: https://doi.org/10.1016/j.epsr.2019.106073.
17. Fekri M, Patel H, Grolinger K, Sharma V. Deep learning for load forecasting with smart meter data: Online adaptive recurrent neural network. Appl. Energy. 2021 Jan 15;282:116177. Available from: https://doi.org/10.1016/j.apenergy.2020.116177.
18. Ozer I, Efe S, Ozbay H. A combined deep learning application for short term load forecasting. Alex. Eng. J. 2021 Aug 1;60(4):3807–18. Available from: https://doi.org/10.1016/j.aej.2021.02.050.
19. Peng J, Kimmig A, Wang J, Liu X, Niu Z, Ovtcharova J. Dual-stage attention-based long-short-term memory neural networks for energy demand prediction. Energy Build. 2021 Oct 15;249:111211. Available from: https://doi.org/10.1016/j.enbuild.2021.111211.
20. Somu N, Raman MRG, Ramamritham K. A deep learning framework for building energy consumption forecast. Renew. Sust. Energ. Rev. 2021 Mar 1;137:110591. Available from: https://doi.org/10.1016/j.rser.2020.110591.
21. Tang Z, Yin H, Yang C, Yu J, Guo H. Predicting the electricity consumption of urban rail transit based on binary nonlinear fitting regression and support vector regression. Sustain. Cities Soc. 2021 Mar 1;66:102690. Available from: https://doi.org/10.1016/j.scs.2020.102690.
22. Somu N, Raman MRG, Ramamritham K. A hybrid model for building energy consumption forecasting using long short term memory networks. Appl. Energy. 2020 Mar 1;261:114131. Available from: https://doi.org/10.1016/j.apenergy.2019.114131.
23. Li R, Jiang P, Yang H, Li C. A novel hybrid forecasting scheme for electricity demand time series. Sustain. Cities Soc. 2020 Apr 1;55:102036. Available from: https://doi.org/10.1016/j.scs.2020.102036.
24. He Y, Zheng Y, Xu Q. Forecasting energy consumption in Anhui province of China through two Box-Cox transformation quantile regression probability density methods. Measurement. 2019 Mar 1;136:579–93. Available from: https://doi.org/10.1016/j.measurement.2019.01.008
25. Bai Y, Xie J, Liu C, Tao Y, Zeng B, Li C. Regression modeling for enterprise electricity consumption: A comparison of recurrent neural network and its variants. Int. J. Electr. Power Energy Syst. 2021 Mar 1;126:106612. Available from: https://doi.org/10.1016/j.ijepes.2020.106612.
26. Eskandari H, Imani M, Moghaddam M. Convolutional and recurrent neural network based model for short-term load forecasting. Electr. Power Syst. Res. 2021 Jun 1;195:107173. Available from: https://doi.org/10.1016/j.epsr.2021.107173.
27. Liu X, Lin Z. Impact of Covid-19 pandemic on electricity demand in the UK based on multivariate time series forecasting with bidirectional long short term memory. Energy. 2021 Jul 15;227:120455. Available from: https://doi.org/10.1016/j.energy.2021.120455.
28. Jana R, Ghosh I, Sanyal M. A granular deep learning approach for predicting energy consumption. Appl. Soft Comput. 2020 Apr 1;89:106091. Available from: https://doi.org/10.1016/j.asoc.2020.106091.
29. He F, Zhou J, Feng Z, Liu G, Yang Y. A hybrid short-term load forecasting model based on variational mode decomposition and long short-term memory networks considering relevant factors with Bayesian optimization algorithm. Appl. Energy. 2019 Mar 1;237:103–16. Available from: https://doi.org/10.1016/j.apenergy.2019.01.055
30. Wang X, Fang F, Zhang X, Liu Y, Wei L, Shi Y. LSTM-based Short-term Load Forecasting for Building Electricity Consumption. In: 2019 IEEE 28th International Symposium on Industrial Electronics (ISIE); 2019 Jun 12–14; Vancouver, BC, Canada. IEEE; 2019. p. 1418–23.
31. Dong Y, Ma X, Fu T. Electrical load forecasting: A deep learning approach based on K-nearest neighbors. Appl. Soft Comput. 2021 Feb 1;99:106900. Available from: https://doi.org/10.1016/j.asoc.2020.106900.
32. Ozdemir S, Susarla D. Feature engineering made easy. Birmingham: Packt Publishing. 2018.
33. Urbanowicz R. An introduction to machine learning [web streaming video]. San Bruno, California: YouTube. 2021 Mar 1 [cited 2022 Jul 1]. Available from: https://youtu.be/K_myCLj2f_Q
34. GeeksforGeeks [Internet]. Uttar Pradesh: GeeksforGeeks; 2022. Feature scaling—Part 3; 2020 Nov 26 [cited 2022 Jul 1]. Available from: https://www.geeksforgeeks.org/feature-scaling-part-3/.
35. scikit-learn [Internet]. [Place unknown]; scikit-learn; 2022. 6.3. Preprocessing data; [publication date unknown] [cited 2022 Jun 30]. Available from: https://scikit-learn.org/stable/modules/preprocessing.html#preprocessing-transformer.
36. Tongta A, Chooruang K. Long short-term memory (LSTM) neural networks applied to energy disaggregation. In: 2020 8th International Electrical Engineering Congress (iEECON); 2020 Mar 4–6; Chiang Mai, Thailand. IEEE; 2020. Available from: https://doi.org/10.1109/ieecon48109.2020.229559

## Automatic process monitoring in a district heating substation utilizing a contextual Shewhart chart

### Henrik Søndergaard^1^, Hamid Reza Shaker^1^, Bo Nørregaard Jørgensen^1^

#### ^1^SDU Center for Energy Informatics, Maersk Mc-Kinney Moller Institute, University of Southern Denmark, Odense M, 5230, Denmark

##### **Correspondence:** Henrik Søndergaard (heso@mmmi.sdu.dk)

*Energy Informatics* 2022, 5(3)

**Summary:** Fault detection methods play a key role in enabling proactive maintenance in district heating systems. Faults are estimated to cause around 40 percent of energy consumption and it is therefore critical to employ methods to decrease this unnecessary waste of energy. For detection of these faults, a data-driven process monitoring methodology is presented which uses a modified version of the Shewhart chart, which is called contextual Shewhart chart. A process variable’s normal operating range often shifts, and this can be due to external factors (e.g., outdoor temperature), and this is not captured by a regular Shewhart chart. However, the proposed contextual Shewhart chart can capture these effects by using a so-called contextual variable to vary the acceptable operational ranges, based on the identified external factor. The methodology has been applied to real data from a district heating substation and has shown promising results.

**Keywords:** Fault detection, Process monitoring, District heating system, Shewhart chart

INTRODUCTION

District heating (DH) can produce and supply heat more efficiently, compared to individual heating in an urban and sub-urban context. As the efficiency is much higher due to e.g., combined heat and power plants [1]. A large proportion of Danish households are supplied by DH, and it has been steadily increasing. Moreover, space heating and hot water use account for a great share of total consumed energy in the EU [2].

Nonetheless, current district heating systems (DHS) are not running optimally. Faults are estimated to cause around 40% of energy consumption in DHS [1], it is, therefore, critical to decrease this figure, to decrease unnecessary expenditure, energy usage, and CO_2_ emissions. According to [3] three-quarters of DH substations display faults, and they are critical in the delivery of heat to customers. Faults do not always lead to a disruption in operation and these faults can therefore remain undetected because the control system can often compensate. This could be a leakage in a pipe, where the flow is simply increased to ensure the supply of heat. Methods for identifying these faults are therefore needed to avoid unnecessary waste.

Process monitoring via the use of fault detection (FD) methods can help alleviate these faults. FD methods have been proven very useful for detecting unusual behavior energy domains.

The application of FD methods in DHS has been reviewed in [1]. FD methods can be subdivided into three main categories. Process history-based, quantitive model-based, and qualitative model-based. The use of model-based methods has proven to be very useful for fault detection in various energy domains, but are often not very generalized and scaleable, but are easily understandable due to the simple and well-known equations utilized. Whereas, process history-based methods can be extremely dynamic, and can capture complex phenomena, that are not easily captured by simple equations [1]. For process history-methods ensuring that the quality of the data is high is an extremely important aspect, and methods for this have been investigated in [4] for sensors in buildings. But in general, there are both pros and cons to the various types of methods. The research focus at the moment largely on process history-based methods in DHS. This coincides with the fact that a large quantity of data is collected in DHS which are not utilized to their full potential. According to [3] sensor readings have the potential to enable proactive maintenance via fault detection methods. The current practice is reactive maintenance which is extremely inefficient.

It is noted in [1] that many of the process history-based approaches are not useful since training the models using simulated or laboratory data does not equate to good performance when applied to real data for testing. However, they are slated to be able to solve the complex FD problems we currently face due to their advantages over model-based methods.

The following small selection of papers shows promising results for FD in DH, but there is still quite a large gap in research [1].

The use of gradient boost regressor for DH substations for FD has been investigated in [5]. Another paper looks at comparing different substation performances via the use of correlation analysis and identifying substations that differs from others as an indicator of faults [6]. They also investigate the use of a limit checking and cluster analysis approach via moving average and standard deviation of the energy usage. Both of these are used to detect faults. The paper [7] uses cluster analysis along with association analysis to decide operational rule patterns of district heating substations. A qualitative evaluation is then used to select significant rules. [8] uses three different clustering methods to identify operational patterns and thereby identify faulty behavior in consumer data.

A Shewhart chart or control chart is a widely researched and utilized method for univariate FD [9], however no research using it in the domain of DH has been identified [1]. Exploring its FD capabilities in DHS is therefore needed. A downfall of regular Shewhart chart is that it is not flexible and dynamic, due to its inherent simplicity. Changing regular operational conditions of systems cannot be captured properly by the static control limits, and a methodology is therefore needed to capture this aspect.

This paper will fill the identified gaps by implementing a modified version of the Shewhart chart, that creates individual/dynamic control limits for observations based on outside ambient temperature observation. This can be called a contextual Shewhart chart, as the sensor readings are put into the context of another variable that is known to affect the system’s operation and uses that fact to define dynamic control limit boundaries. For example, energy consumption of a system of 14 MWh for an hour is normal during the winter with low ambient temperatures, but not during the summer with higher ambient temperatures. A regular Shewhart chart is not able to properly account for this complexity, as it assumes the process does not change normal operational behavior over time. The methodology will be applied to real data from a DH substation, which according to [3] have a very high proportion of faults. This, therefore, fills the gap/issue raised regarding simulated/laboratory data.

A similar methodology has been developed in [10], where the control limits are based on a piecewise linear regression given a contextual variable. The methodology developed in this paper is more flexible, because one does not need to manually inspect the data to determine the cutoffs for the “pieces”, and the control boundaries are more dynamic. The method in this paper can therefore be seen as a sort of improvement of the method in [10].

To summarize, the contributions of this paper is the usage of real-world data, which includes a known fault for evaluation of the method. On top of this, the methodology proposed in this paper is a novel method and has therefore not been applied before in DHS.

The first section of the paper will discuss in more detail the methodology of the contextual Shewhart chart. Then a case study is introduced and lastly application results for the case study.

METHODOLOGY

Ordinary Shewhart Chart

Shewhart charts or control charts are a univariate statistical process monitoring method that can be used for determining if a process variable is outside the regular operating range, based on upper and lower control limits [9]. The control limits (CL) can be determined by healthy training data and are calculated as seen in Eq. 1. The equation assumes only one control variable.

1 $$\text{CL}=\overline{x}\pm r\cdot \text{S},\;\text{ where}\;S=\sqrt{\frac{{\sum }_{n=1}^{N}{\left({x}_{n}-\overline{x }\right)}^{2}}{N-1}}\; and \; \overline{x }=\frac{{\sum}_{n=1}^{N}{x}_{n}}{N}$$

where $\overline{x}$ is the sample mean of the training data, $\text{S}$ is the sample standard deviation of the training data, and $r$ is the number of standard deviations, and $r\cdot \text{S}$ is the confidence interval. Where for $r = 3$, 99.7% (level of significance) of the training data would be within the control limits, given a normal distribution. Choosing an $r$ value is a trade-off between missed detections and false alarm rates. Increasing the $r$ value decreases the false alarm rate and increases missed detections, and vice versa. The control limits can then be applied to potentially faulty test data. When an observation for the control variable is outside the control limits an alarm is raised, indicating that a fault is present. This can be visualized in Figure 1.


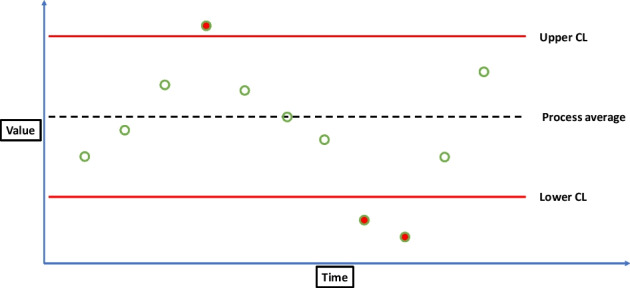


Figure 1 Visualisation of a Shewhart chart. Red filled in circles are observations outside control limits which raise alarms

Modified Control Chart

A regular Shewhart chart is a quite straightforward methodology to implement because the control limits are static, and for many processes, this might be satisfactory. However, some process variables in some domains, normal acceptable ranges change/shift, based on external factors such as outdoor temperature or modified setpoints. This would change the mean, $\overline{x }$, for the observations after the shift, and the control limits should therefore shift. This would not be correctly reflected in a regular Shewhart chart. A modified version of the the control chart is therefore needed to account for such changing conditions and the resulting acceptable behavior. One could do a rolling Shewhart chart, with the limits based on the last $n$ observations’ mean and standard deviation. However, the methodology in this paper uses the identified external factor as a way to create dynamic control limits.

The modified Shewart chart presented in the following section creates individual control limits based on a contextual variable. It can therefore be called a contextual Shewhart chart. The structure of the input data can be seen in Eq. 2. Where $x$ is a matrix containing the sensor data, with $j$ number of process variables and $N$ observations. $C$ contains the so-called contextual sensor variable, which will be used to categorize the different observations in $x$. ${C}_{1}$ is measured at the same time as ${x}_{11}\cdots {x}_{1j}$. Each row in $x$ is an observation vector.

2 $$x=\left(\begin{array}{cccc}{x}_{11} & {x}_{12} & \cdots & {x}_{1j}\\ {x}_{21} & {x}_{22} & \cdots & {x}_{2j}\\ \vdots & \vdots & \ddots & \vdots \\ {x}_{\text{N}1} & {x}_{\text{N}2} & \cdots & {x}_{Nj}\end{array}\right), \; C=\left(\begin{array}{c}{C}_{1}\\ {C}_{2}\\ \vdots \\ {C}_{N}\end{array}\right)$$

The proposed methodology for training is described in Table 1. For testing, only step 4) is applied to the new data. The control limits determined by the training methodology are then applied to the sorted test data. The manner in which the results are visualized can help with the understanding of the methodology, and the following section describes that aspect.

Table 1 Methodology overview. Creation of model (training)

**Table:** 

1. Determine the contextual variable and sensors to monitor from inspection of cross correlation plots. The sensors that are appropriate for the methodology have either strong negative or positive correlation with a given contextual variable
2. Create intervals of the contextual variable from its maximum value to minimum value with certain interval size. Resulting in $k$ intervals or bins. The term “intervals” will be used in this paper. (See discussion in results section for determining the interval size)
3. Create $k$ number of empty matrices. Where the first matrix is associated with the first temperature interval, the second matrix for the second etc.
4. Sort each observation vector in $x$ into the appropriate matrix according to what interval the corresponding observation value in $C$ belongs to
5. For each matrix containing various number of observations, calculate the mean and standard deviation and thereafter the upper and lower control limits according to Eq. 1. This is done for each individual process variable

Figure 2 is an example of a way to display the contextual Shewhart chart for a return temperature sensor in a district heating pipe on the y-axis. Outdoor temperature intervals along the x-axis where the blue dots are test data. The upper and lower control limits are red, and the mean is the dashed line based on the training data. Figure 3 shows a more regular Shewhart chart but with control limits changing over time due to the different outdoor temperature intervals that apply to each observation. It is a cutout from the whole data series.

Some sensors can be labeled as so-called “zero sensors”, which observations are either at some significant mean value, or is at zero for a certain extent of time, these do not work well with the current methodology as these zero observations could skew the control limits significantly. An example of such a sensor could be a heat production unit that often shuts completely off or is hovering at a steady heat output otherwise. Control limits are only wanted for the periods the production unit is in use and only be based on training data for when the unit is in use, for the given contextual variable intervals. The methodology is thereby changed for the identified zero-sensors to training with only the non-zero values and not labeling zero observations as faults in testing.
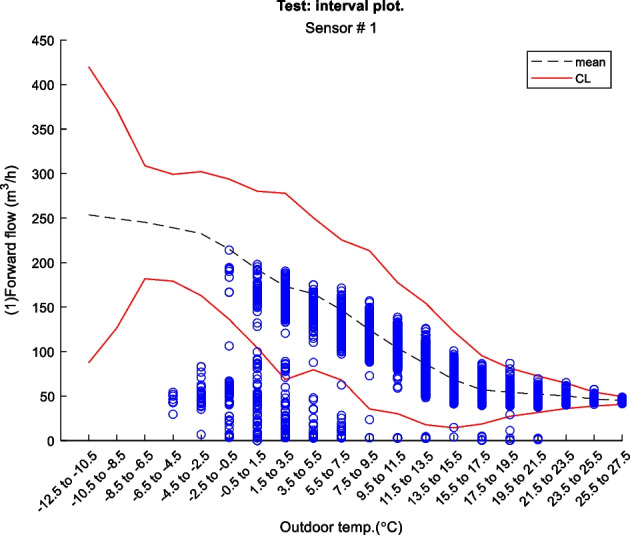


Figure 2 Contextual Shewhart chart example
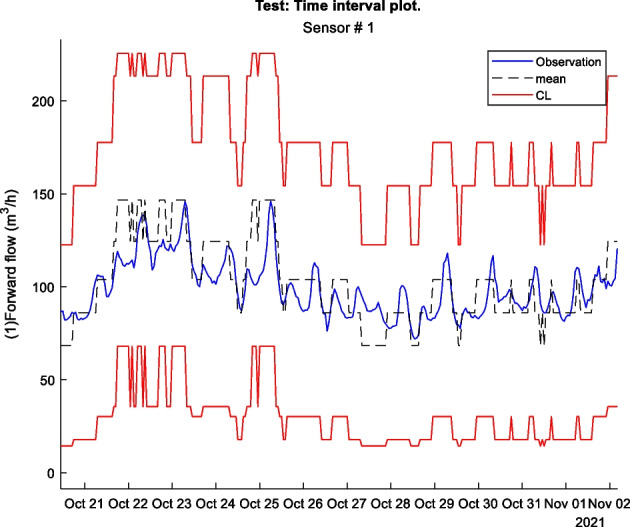


Figure 3 Contextual Shewhart chart example 2. NOTE: only shows part of the data from Figure 2

Description of Case Study and Faults

Time series sensor data from a pumping substation in Odense, Denmark will be used for the application of the methodology. The data is from 16 sensors and is measured on an hourly basis for the year of 2021. A description of the different sensors can be seen in Table 2 and a general visual representation of the placement of a selection of the sensors at the substation in Figure 4. Sensors 4 and 5 are critical pressures at two nodes in the distribution system. The outdoor temperature will be used as the contextual variable (it is therefore not numbered). It is chosen based on the fact there is seasonal variability in the system’s operation, and the outdoor temperature is a good indicator for capturing this aspect. However, the use of other sensors as the contextual variable is also viable, but will not be showcased in this paper. More in-depth inspection of the cross-correlation plots are in the results section.

Table 2 Description of sensors. Zero sensors are marked red. RP = return pump

**Table:** 

#	Sensor description	#	Sensor description
(1)	Forward flow (${\text{m}}^{3}/\text{s}$)	(9)	Return pressure after RP1(bar)
(2)	Forward temp. (°C)	(10)	Return pressure before RP1(bar)
(3)	Forward pressure (bar)	(11)	Pressure after holding valve (bar)
(4)	Forward pressure (NLM) (bar)	(12)	Forward temp. (°C)
(5)	Forwards pressure (ROM) (bar)	(13)	Forward pressure (bar)
(6)	Mixing flow (${\text{m}}^{3}/\text{s}$)		Outdoor temperature (°C)
(7)	Return flow (${\text{m}}^{3}/\text{s}$)	(14)	Local production (MWh)
(8)	Return temp. (°C)	(15)	Forward energy (MWh)


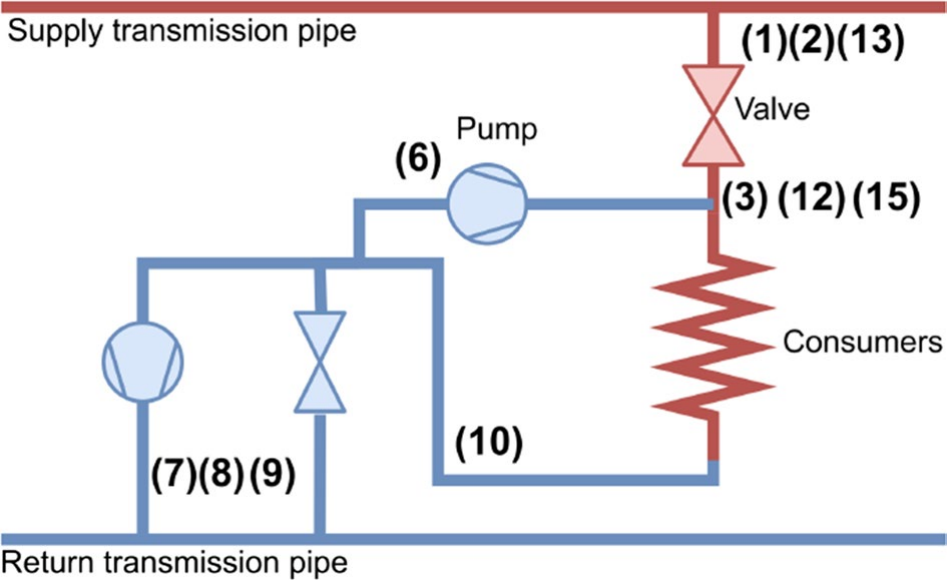


Figure 4 Visualisation of the substation and placement of sensors

In the data, a known fault exists for forward energy (15). This value was miscalculated (it is therefore not a physical sensor). This was due to a scaling error of the forward flow (1) in the smart meter. This resulted in the forward energy being lower than it should have been. Therefore, for sensor 15 there is both data available with the fault and data without the fault (because it was properly calculated after the fault was detected manually). It is therefore possible for that sensor to apply the methodology for the training with the healthy data and testing with the faulty data. A visual representation of the correct and incorrect data for the testing data can be seen in Figure 5 along with the residual error.
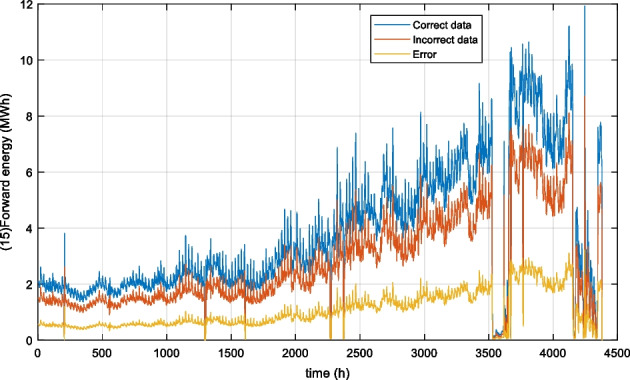


Figure 5 A visual representation showing the known fault (red line) in the test data and the residual error

An important aspect that must be brought forth is the notion that there might be faults present in the training data. The most optimal scenario would be that the training data was fault-free, and encapsulates the complete regular operational spectrum. The latter aspect is partially dealt with by including both winter and summer data in the training data. The data from the first half of 2021 will be utilized for training while the latter half will be used for testing.

RESULTS

Firstly, an overall summary of the faults from the various sensors will be presented, along with setting parameters for the methodology. Thereafter, more in-depth discussions regarding fault detection for a few sensors will be presented.

General Results and Parameters

As discussed in the previous section, the choice of contextual variable has been picked, however, the reason behind this can be visualized in Figure 6, in which it is apparent that there is a strong correlation between the sensor displaying a known fault (15) and the contextual variable outdoor temperature. It is not the strongest correlation for sensor 15, however, a more interesting application example as it is an external variable, compared to utilizing forward flow, which has a cross-correlation of 1. Other potential candidates for a contextual Shewhart chart using outdoor temperature as the contextual variable are sensors 1, 2, 6, 7, 9, and 12 as all have negative correlations of below − 0.9, while 10 and 11 are 0.9.
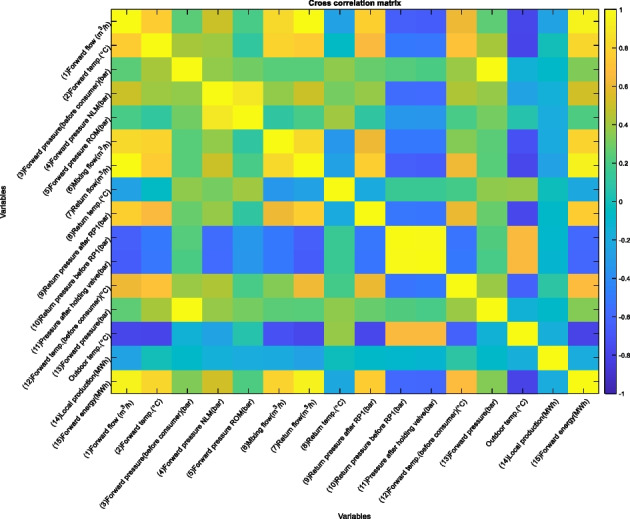


Figure 6 Cross correlation plot

Firstly, an interval size has to be picked for the contextual variable. Too low values result in too few observations to train for each interval. A too large value is not desired either, because it defeats the purpose of the method and becomes too close to a regular Shewhart chart. A balance was struck at 2 °C through trial and error.

Figure 7 displays the general fault rates for the different sensors, however as mentioned, it is only interesting to look at the sensors which have the highest correlation with the contextual variable.

Operational parameters can change over time. This could be a setpoint for the forward temperature, this adjustment can result in the testing data displaying a significant number of faults. These changes inhibit the methodology from working properly, as they need continuous retraining due to changes in setpoints or other changes in system configuration. This issue may also be present in the following data, as these changes could happen throughout the year. The significant alarm rates for sensor 3, 4 and 13 are mainly due changes in operational behaviour.

The extreme temperature intervals do not include many observations for training, and the alarms raised in testing for those intervals are not as robust as the intervals with more training data.
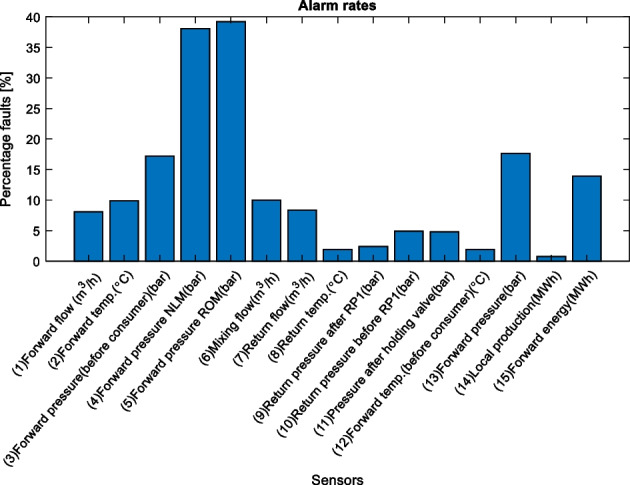


Figure 7 Fault rates for all sensors using outdoor temperature as the contextual variable

In-Depth Fault Results

Firstly, the sensor with the known fault will be discussed, to determine if it was able to detect the fault. It can be seen from Figure 8 that the overall test data is lower than the mean for each temperature interval, which falls in line with the expected result. And thereby a significant portion is outside the control limit. However, an inspection of Figure 10 is needed for more context, where the most severe alarms are visualized. The reason for these large drops in forward energy seen in the figure is due to local production, and it is therefore not attributed to the known fault. This behavior is not easily captured by the model. The magnitude of the fault is not large enough at most times for the method to detect it. A change in the confidence interval to $2\sigma$ is carried out to see how this affects the alarm rate and can be visualized in Figure 9. The alarm rate increases from 14 to 31%, and it detects the known fault to a larger extent.

The interval plot for sensor 1 from the methodology section (Figure 2) also shows quite a significant number of observations under the control limit, and this is solely due to the local production, as flow from the transmission grid is not needed to the same degree when there is local production.
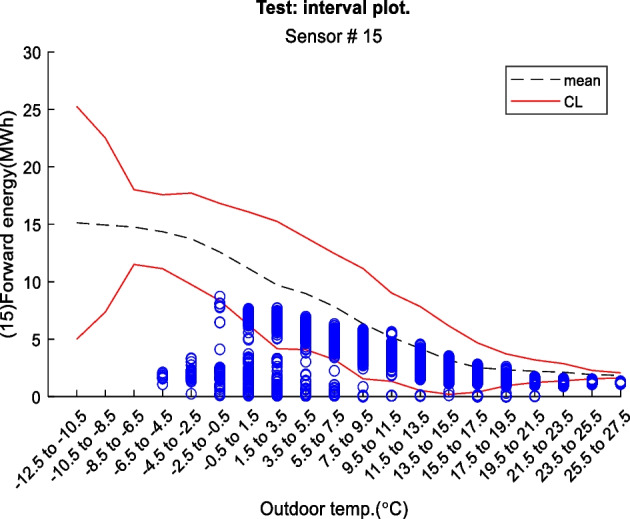


Figure 8 Shewhart chart interval plot for forward energy (15) with confidence interval of $3\sigma$
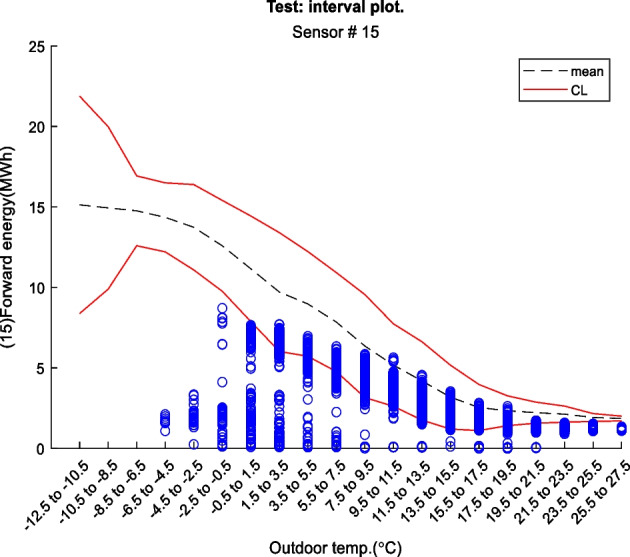


Figure 9 Shewhart chart interval plot for forward energy (15) with confidence interval of $2\sigma$
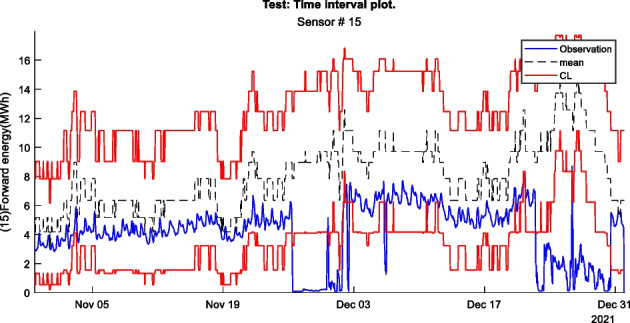


Figure 10 Partial Shewhart chart for forward energy (15) with confidence interval of $3\sigma$. NOTE: does not show the whole testing period

A possible example of changes in operational parameters for the system is for sensor 2. The training and testing Shewhart chart interval plot can be seen in Figure 11 and Figure 12, respectively. A general overall decrease in forward temperatures for all intervals is observed, and this can be due to a decrease in setpoint temperature. However, the contextual Shewhart chart can capture the underlying dynamics of the system with larger allowed forward temperatures for lower outdoor temperatures. To reiterate, retraining of the model is needed when e.g., a setpoint changes, as the regular operating range could shift.
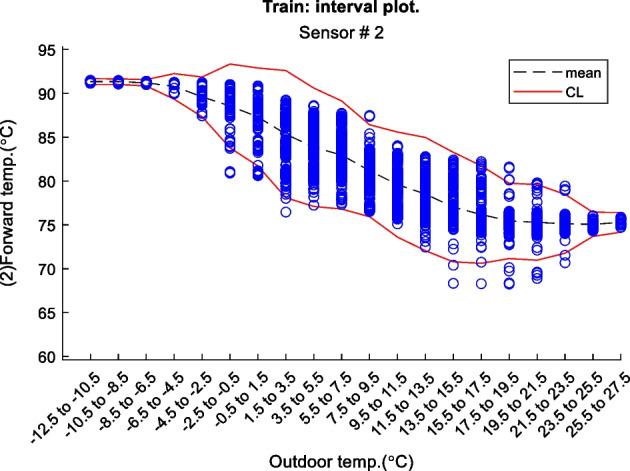


Figure 11 Training Shewhart chart interval plot for forward temp. (2)
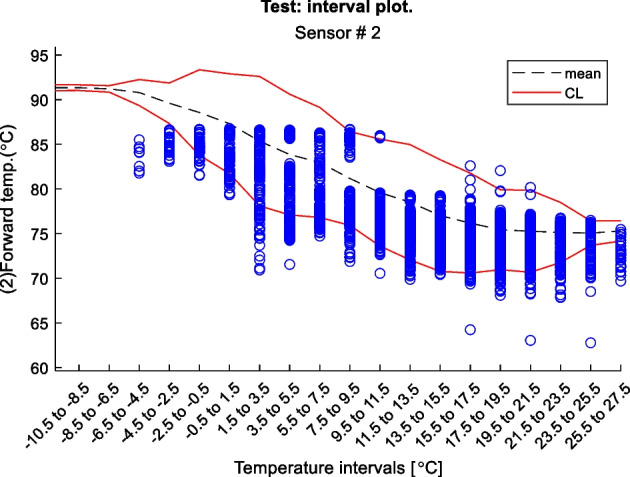


Figure 12 Testing Shewhart chart interval plot for forward temp. (2)

Lastly, an example of what a resulting Shewhart chart looks like when the correlation is not strong. Sensor 8 is the sensor with almost the lowest detected fault rate. The training and testing Shewhart chart interval plot can be seen in Figure 13 and Figure 14, respectively. The correlation between the two variables is 0.65 and is therefore not a strong correlation. This equates to quite significant standard deviations for each of the intervals and the mean is also quite similar (flat) for each of the intervals. Most observations in testing are thereby within the control limits due to the large standard deviation for each interval in training. Applying this methodology to this sensor is therefore not as useful. The contextual variable cannot/does not capture a general tendency/trend in the data.
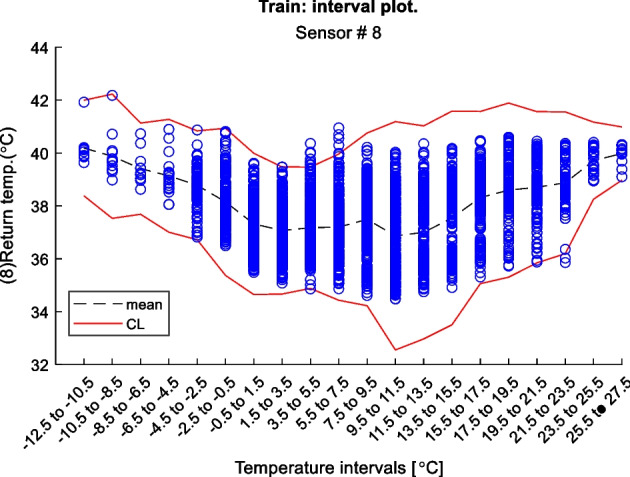


Figure 13 Training Shewhart chart interval plot for return temp. (8)
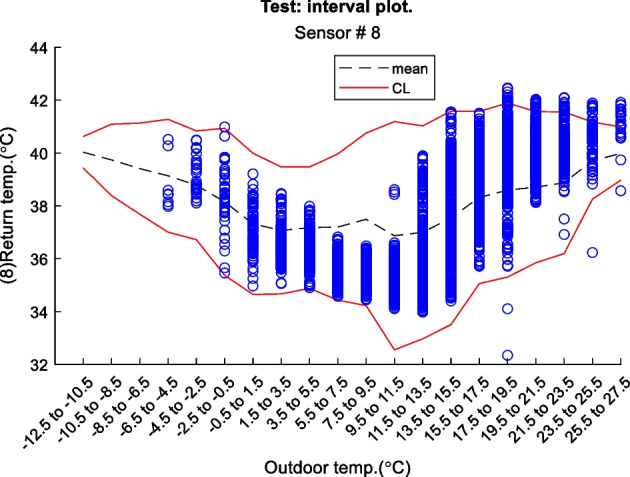


Figure 14 Testing Shewhart chart interval plot for return temp. (8)

The contextual Shewhart chart can be compared and is slightly related to machine learning prediction, in which the contextual variable is the independent variable in this case. Instead of fitting with e.g., linear regression which is continuous (whereas this methodology is not), this methodology separates the independent variable into different intervals and uses the mean of the dependent variable from training in each of the intervals as the prediction.

CONCLUSION AND FUTURE WORK

The proposed contextual Shewhart chart methodology has been implemented on real data from a district heating substation, and it has proven to be able to detect a known fault, with some adjustment of control limits. In general, it has displayed its capabilities in being able to better capture normal operational trends and system behavior for the various process variables. It is therefore a significant improvement over regular Shewhart charts for systems in which normal operational behavior changes based on external/internal factors, such as outdoor temperature. Limitations. A downfall of the methodology is that it is does not work well with changes in set points, as they can result in vastly different normal acceptable operational behaviour. The model needs to be retrained to take into account such a change. The methodology is easy to implement for any number of sensors and is very dynamic in nature.

Future work includes being able to determine the severity of the faults, as the rate of faults is so significant that it is not possible to assess them all. To increase the confidence that a fault is a true positive, multiple FD methods could be employed. On top of this, more testing with known faults from real-world data or manually induces fault is needed for validation of the methododology.

**List of abbreviations**

- DH: District heating
- DHS: District heating system
- FD: Fault detection
- CL: Control limit

**Acknowledgements:** This paper is part of the funded project “Proactive and Predictive Maintenance of District Heating Systems” by the funding agency Danish Energy Agency under the Energy Technology Development and Demonstration Program, Denmark. ID number: 64020–2102.

**References**

1. Buffa S, Fouladfar MH, Franchini G, Lozano Gabarre I, Andrés Chicote M. Advanced Control and Fault Detection Strategies for District Heating and Cooling Systems—A Review. Applied Sciences. 2021;11(1):455.
2. EU. “Commission recommendation (eu) 2019/786 of 8 may 2019 on building renovation. 2019.
3. Gadd H, Werner S. Fault detection in district heating substations. Applied Energy. 2015.
4. Mattera CG, Lazarova-Molnar S, Shaker HR, Jørgensen BN. A Practical Approach to Validation of Buildings’ Sensor Data: A Commissioning Experience Report. 2017 IEEE Third International Conference on Big Data Computing Service and Applications (BigDataService)2017. p. 287–92.
5. Månsson S, Kallioniemi P-OJ, Sernhed K, Thern M. A machine learning approach to fault detection in district heating substations. Energy Procedia. 2018;149:226–35. https://doi.org/10.1016/j.egypro.2018.08.187.
6. Sandin F, Gustafsson J, Eklund R, Delsing J. BASIC METHODS FOR AUTOMATED FAULT DETECTION AND ENERGY DATA VALIDATION IN EXISTING DISTRICT HEATING SYSTEMS. 2012.
7. Xue P, Zhou Z, Fang X, Chen X, Liu L, Liu Y, et al. Fault detection and operation optimization in district heating substations based on data mining techniques. Applied Energy. 2017;205:926–40. https://doi.org/10.1016/j.apenergy.2017.08.035.
8. Sun W, Chen D, Peng W. Anomaly Detection Analysis for District Heating Apartments. Journal of Applied Science and Engineering. 2018.
9. L.H C, E.L. R, R.D. B. Fault detection and diagnosis in industrial systems. Springer-Verlag London Ltd; 2001.
10. Månsson S, Davidsson K, Lauenburg P, Thern M. Automated Statistical Methods for Fault Detection in District Heating Customer Installations. Energies. 2019;12(1):113.

## A probabilistic approach to reliability analysis of district heating networks incorporating censoring: a report of implementation experiences

### Lasse Kappel Mortensen^1^, Hamid Reza Shaker^1^, Christian T. Veje^1^

#### ^1^SDU Center for Energy Informatics, Maersk Mc-Kinney Moeller Institute, University of Southern Denmark, Odense SØ, 5230, Denmark

##### **Correspondence:** Lasse Kappel Mortensen (lkmo@mmmi.sdu.dk)

*Energy Informatics* 2022, 5(3)

**Summary:** Reliability analysis has the potential to provide actionable insight into the failure probability of assets in district heating networks. Information about the failure rate and its trend may help operators and asset managers replace assets at the optimal time, which can increase the security of supply, save resources, and reduce operational and maintenance costs. In this paper, we employ a probabilistic proportional hazard modeling approach to reliability analysis, which has not been used for district heating pipes before, explore its potential and report our experiences. The model allows us to model the time-dependent survival probability of pipe assets as a function of asset-related and environmental predictors which have been shown to influence failure probability in previous studies. We find that the application of the model in this domain is challenged by several issues pertaining to data, one of which we attempt to remedy with a simple imputation strategy.

**Keywords:** Reliability analysis, District heating, Predictive maintenance, Data collection

INTRODUCTION

Breaks and leaks in district heating systems can cause losses of tens to hundreds of thousands of cubic meters of water yearly. Aside from water being expensive to replace, breaks in district heating pipes can decrease the supply to a level where consumers do not have access to the heat they require. While repairing breaks is expensive, pre-emptive replacement of pipes is inefficient in terms of both cost and other resources. Determining when the optimal time is to replace a pipe requires information about the expected time to failure, frequency of failure, time-dependent survival probability, or similar. “Reliability” or “survival” analysis sets out to determine exactly this optimal replacement time.

Survival analysis is a branch of statistics that is native to the medical industry but is also used (under other names) in other disciplines e.g., in the engineering domain it is referred to as reliability analysis. Purely physics-based models are rarely used to find the expected time to failure because they are very time-consuming to implement and can have unrealistic requirements for sensor input. Nevertheless, if the sensor data is available, physics-based degradation models have very high accuracy.

A common alternative to physics-based models is statistical models, which can be roughly divided into deterministic and probabilistic models. Deterministic models employ regression techniques such as least-squares regression to fit e.g., a Poisson-generalized linear regression model or a multivariate linear regression model [1, 2]. The probabilistic alternative to statistical modeling uses e.g. Monte Carlo simulation or Bayesian statistics [3–5], the former potentially including methods for Markov Chain Monte Carlo simulation of the conditional density of regression coefficients [3].

Rimkevicius et al. developed a methodology for assessing reliability of energy networks which was applied to a district heating network. The method consisted of both deterministic and probabilistic modelling elements and was able to identify the most failure-prone pipe sections and to estimate failure consequences at the consumer level [6]. Postnikov et al. used Markov random process theory to model the reliability of systems of district heating assets [7]. The simulated reliability study of combined assets showed that the reliability of district heating systems is mostly affected by individual assets with poor reliability. Valincius et al. used simple statistical analysis to identify failure-prone pipes in a district heating network and subsequently deterministically model only the most failure-prone pipes [8].

Event time information in survival data is rarely complete. E.g. a pipe can have been broken for several months before it is noticed, in this case the actual time the pipe broke is unknown, which can be referred to as incomplete event time information. This phenomenon biases survival models. To reduce the bias some probabilistic survival models account for incomplete event time information using censoring [3–5, 9, 10], which will be explained in more detail later in this paper.

A sub-category of probabilistic models referred to as Proportional Hazard Models (PHM) are unique in that they explicitly parameterize the effects of a set of covariates on survival time. These covariates typically explain the material type, nominal dimensions, etc. when employed for pipes [4, 5].

Recently there have also been some applications of machine learning for survival analysis of pipes, e.g., [2] in which a neural network was used for binary classification and remaining-life prediction. Recently, a combination of survival modeling and machine learning involving random survival forest techniques was applied to survival analysis of water pipes [11]. Random Forest considers the log-rank statistics between cumulative hazard functions of child nodes when performing splits, which means the model can be used on censored data.

In recent work on relative fault vulnerability prediction, our results corroborated that the environment surrounding district heating pipes seems to affect their failure rate [12]. These environmental features can be integrated into the survival modeling of district heating pipes using the PHM approach, as has been done for water distribution systems [1].

In this paper, we implement the censor-adjusted Weibull Proportional Hazards Model (WPHM) in the district heating domain which, to the best of the authors' knowledge, has not been done before. We use the PHM approach to model environmental lying conditions’ effects on district heating pipes. We explore the challenges that arise from working with a relatively new district heating network, i.e., a network in which only a small fraction of the pipes has reached the wear-out stage of their lifecycle. Furthermore, we consider the potential influence of suboptimal data collection, discuss the use of imputation, and suggest several criteria for good data concerning survival analysis.

The paper is structured as follows: First, we explain the methodology of applying the survival model, then we introduce the case study to which survival model is applied. Lastly, we present and discuss the results, emphasizing finding reasonable explanations for the discrepancies that we observe between the values predicted for life expectancy of district heating pipes according to our model and industry belief.

RELIABILITY MODEL FOR MAINTENANCE OF DISTRIBUTION SYSTEMS

In this section, the theory behind the probabilistic survival analysis employed in this paper and its application is introduced. Kabir et al. [3] present a summary of survival analysis methods applied to water distribution systems. They find that the exponential model, Weibull model, Cox proportional hazard model (cox-PHM), and the Weibull proportional hazard model (WPHM) are particularly well-regarded and widely employed. The proportional hazard models share an interesting characteristic, in that they integrate a predictor term in their hazard function that depends on a set of covariates, meaning they can model the correlation between, e.g., pipes’ lying conditions or asset information and their reliability directly [13].

The cox-PHM is described in [14]:

1 $$h\left(t|X\right)={h}_{0}\left(t\right){e}^{X\beta }$$

where $h(t|X)$ is the time- and covariate-dependent hazard function, ${h}_{0}$ is the baseline hazard function, $t$ is time with reference to when the study period begins, and $X$ and $\beta$ are the covariates and the covariate coefficient vectors, respectively. From (1), it can be seen that the second factor is independent of time, i.e., the covariates’ effect on the baseline hazard function does not change over time [4]. The survivor function is given by:

2 $$S\left(t|X\right)={S}_{0}\left(t\right){e}^{X\beta }$$

where ${S}_{0}$ denotes baseline survivor function.

In the WPHM, the survivor function is given by [15]:

3 $$S\left(t|X\right)={e}^{-{e}^{\left(\frac{{\log}\left(t\right)-\mu -X\beta }{\sigma }\right)}}$$

where $\mu$ is a constant called intercept and $\sigma$ is a scale parameter. The WPHM is derived from the log-linear relations [15]:

4 $${\log}(t)= {\mu} + {\beta X} + {\sigma \epsilon}$$

where $\epsilon$ is an error term. The log-linear relationship shows that there is an interaction between the covariates and time in the WPHM which is not present in the cox-PHM. This is one reason the WPHM is gaining more attention [3]. For this study, we also use WPHM.

The parameters for the WPHM can be estimated using maximum likelihood estimation, the likelihood function of the WPHM is given by:

5 $$L(\beta ,\mu ,\sigma |data)= \prod_{i=1}^{n}{f}_{i}{\left({\log}({t}_{i})\right)}^{{\delta }_{i}}{S}_{i}{\left({\log}({t}_{i})\right)}^{1-{\delta }_{i}}$$

where ${\delta }_{i}$ is a censoring indicator and ${f}_{i}$ is the density of the log time, given by

6 $${f}_{i}\left({t}_{i}\right)=\frac{1}{\sigma }{e}^{\left(\frac{{\log}\left({t}_{i}\right)-\mu -{X}_{i}\beta }{\sigma }\right)-{e}^{\left(\frac{{\log}\left({t}_{i}\right)-\mu -{X}_{i}\beta }{\sigma }\right)}}$$

where *i* is an index ordinal denoting a specific observation.

Censoring

Figure 1 illustrates the concept of censoring. Since most of the pipes in the case study have not failed yet, the observations in the data are predominately right-censored, meaning that the time, ${t}_{i}$, of a right-censored observation can be considered to be the pipe’s minimum lifetime. Since the thermography measurements are not performed continuously but at regular intervals, when a fault is observed, the actual time of the fault is anywhere between the time of observation and the time of the previous observation of the pipe. This should be treated with interval-censoring. Pipes that have failed and been replaced without any record are omitted from the study and the dataset is therefore defined as left-truncated. Pipes that are currently installed and have failed before observations began should be left-censored since the fault has happened before *t* = 0. In reality, faults have been repaired before the start of the study period, which means that pipes that should have been left-censored can only be represented as right-censored considering the available information.
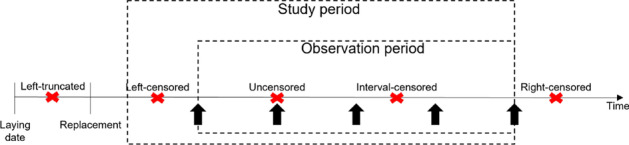


Figure 1 Illustration of types of censoring and truncation typically encountered in survival analysis. The black arrows represent observations, and the crosses represent faults

The likelihood function, adapted to account for interval censoring, expressed as only right, and interval-censored observations can be described as [16]:

7 $$L(\beta, \mu, \sigma |data)= \prod_{i=1}^{n}{S}_{i}{\left({\log}({t}_{i})\right)}^{{\delta }_{i}}\prod_{i=1}^{n}{\left({S}_{i}\left({\log}({t}_{i,lb})\right)-{S}_{i}\left({\log}({t}_{i,ub})\right)\right)}^{1-{\delta }_{i}}$$

where ${\delta }_{i}=1$ denotes right censoring and ${\delta }_{i}=0$ denotes interval censoring. ${t}_{i,lb}$ and ${t}_{i,ub}$ denotes the lower and upper bound for the survival time of pipe $i$ respectively, assuming the pipe failure is immediately noticeable. The left product, therefore, pertains to right-censored observations only, and the right product pertains only to interval-censored observations.

Posterior sampling

To maximize insight into the parameters of the posterior distribution, maximum likelihood estimation (MLE) is first used to get an initial estimate of the parameters. Subsequently, samples are taken from the posterior distribution:

8 $$f(\sigma, \mu, \beta |Data)$$

using the Metropolis–Hastings random walk algorithm [15], because of its efficiency and popularity [17]. This enables studying the uncertainty of the parameter estimates of the survivor model. We use a normal proposal density, define by a mean vector of zeros and the variance–covariance matrix of the parameters $\sigma$, $\mu$, $\beta$. As suggested by [15] we set the scale parameter of the proposal density so that the acceptance rate of simulated draws is in the range 20–40%.

In summary, an asset’s survival probability is calculated using (3), the parameters of which are determined using the Metropolis–Hastings random walk algorithm, with the initial parameter estimate determined using the maximum likelihood estimation of (7). We use the Metropolis–Hastings random walk algorithm to simulate 10,000 draws from the posterior density (8), meaning the parameters of the model given in (3) given a specific dataset. Predictions are obtained by solving (3) for each simulated draw of the posterior density (7, 8) and characterizing the statistical distribution of the output survival probability.

CASE STUDY

We implemented the reliability model using data from a district heating network on the island of Funen, which represents Danish district heating pipe networks well. The network supplies more than 100,000 consumers and consists of more than 140,000 pipes. The pressure ranges from < 25 bar in the transmission pipes to < 6 bars in the distribution pipes. The dataset covers a span of 5 years.

In a previous study [12] we created a dataset based on the geographic information system (GIS) of the district heating system, its historical maintenance record, and relevant GIS data representing the external environmental lying conditions of the pipes. Detailed information about the environmental datasets is given in [12]. The raw datasets were retrieved from [18–20]. The environmental conditions give insight into the chemical and mechanical stresses the pipes may be exposed to.

Using this dataset, in [12] we predicted number of faults and ranked the pipes according to their relative vulnerability. Using our ranking method on this dataset showed that 30% of the network was responsible for 60% of the historical faults in the test data. In this paper, we use a subset of that dataset consisting of the 8 most important, non-redundant, features. These are listed in Table 1.

Table 1 Descriptions of selected features

**Table:** 

Feature	Description
Number of joints	Number of joints along a pipe section
High-risk area	Whether a pipe is located in an area with a high failure risk
Nominal dimension	The nominal internal dimension of a pipe
5 Meter minor road proximity	The proportion of a pipe that is closer than 5 m to a minor road
Level	Ordinal encoding of the hierarchical classification of a pipe, e.g., transmission level or distribution level
Mean redox depth	The average depth to anaerobic soil conditions along a pipe section
1 Meter track proximity	The proportion of a pipe that is closer than 5 m to a track
DSG type soil coverage	The proportion of a pipe that is located in meltwater-sand and -gravel

RESULTS AND DISCUSSION

This section presents results relevant to the evaluation of the survival model, comparing the model with results from our previous work [12]. The median survival probability as a function of time for the entire population of pipes is investigated and compared with life expectations by the Danish district heating association. Lastly, we explore the distribution of age and faults in the data, and test an imputation method to identify the reason for the discrepancy between predictions of expected lifetime according to our model and industry belief.

Figure 2 shows the survival probability for the most and the least at-risk pipe according to our previous study [12]. The plots are produced by calculating the survival probability, using (3), at various times for each simulated draw of parameters given by the posterior density (8).
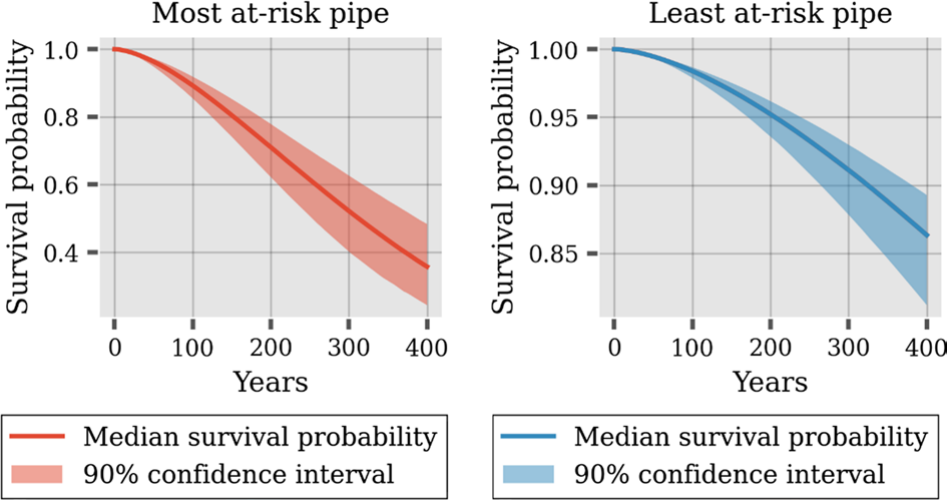


Figure 2 Survival probability, calculated using the WPHM with simulated draws of its parameters, for the most at-risk pipe (red) and the least at-risk pipe (blue) according to our previous study. The lines denote median survival probability, and the areas denote 90% confidence intervals

Both pipes seemingly have incredible long lifetimes, with expected life being approximately 300 years for the most at-risk pipe and well past 400 years for the least at-risk pipe, here expected life is define as the time where the survival probability falls below 50%. This is not aligned with experts’ knowledge, which is that the pipes likely will not survive hundreds of years. The Danish district heating association, “Dansk Fjernvarme”, claims that the expected lifetime of Danish district heating networks is 50–100 years [21]. The Danish district heating association claim 50–100 years is a long expected lifetime and that it is in part credited to the relatively low temperatures that Danish district heating networks are operated at, 70–80 degrees celsius.

Figure 3 visualizes all pipes’ predicted median survival probability as a function of service life. According to the WPHM, the expected median survival probability at 400 years is close to 90%. This emphasizes the order of magnitude for the difference between the expected life according to the model the expectation of industry experts.
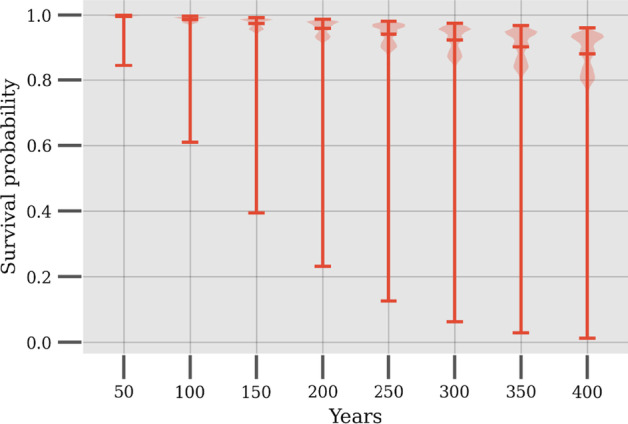


Figure 3 Violin plot of median survival probability of the entire population of pipes at different service lives. The horizontal lines represent max (top), median (middle), and min (bottom) median survival probability

Three reasons, in particular, can explain why the observed trend deviates so much from what is expected. Firstly, the maintenance record, i.e., thermographic imaging in this case, only spans 5 years, while the oldest pipes are more than 40 years old, see Figure 4. The data is in that sense, predominately left-censored, which results in a discrepancy between the observed number of failures and the actual number of failures, with actual failures including failures that happened before the study period. This biases the model towards a longer survival time, as pipes that broke and were repaired or replaced before the study period began are not represented with accurate failure times. Since it is unknown which pipes broke and were repaired before the study period began, the pipes that should be left-censored are represented as right-censored observations, and pipes that have been replaced before are truncated.
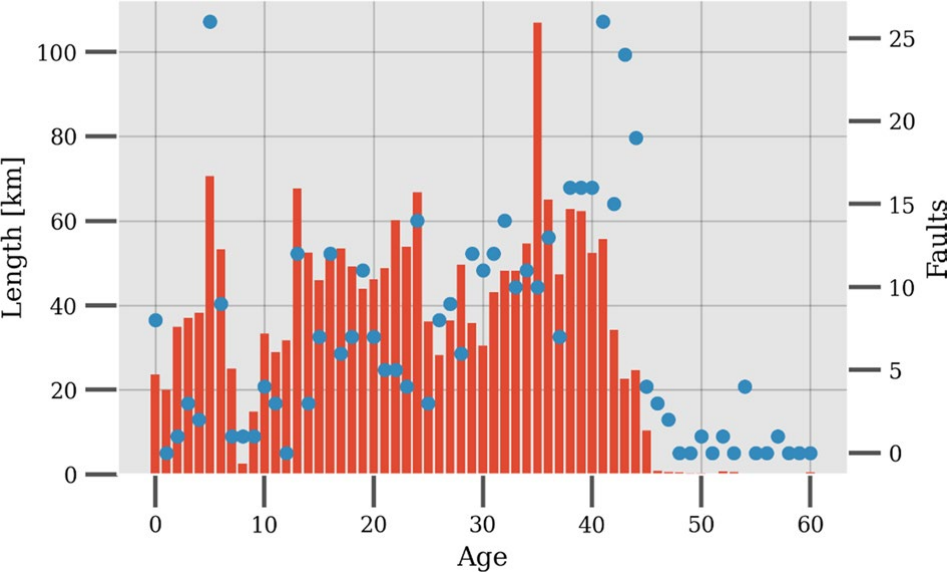


Figure 4 Histogram of the age distribution of the district heating pipes. The blue dots denote the number of historic faults for pipes in that age bin

To study the magnitude of the bias introduced due to left-censoring, we employ a basic imputation strategy and reevaluate the parameters of the WPHM. The imputation strategy makes use of the observed time-dependent failure rate, visualized in Figure 5. The figure shows the failure rate as a function of age. Since the fault observations span multiple years, note that the individual pipes at a specific age change from year to year. The histogram, therefore, shows the average length of pipes at each bin.
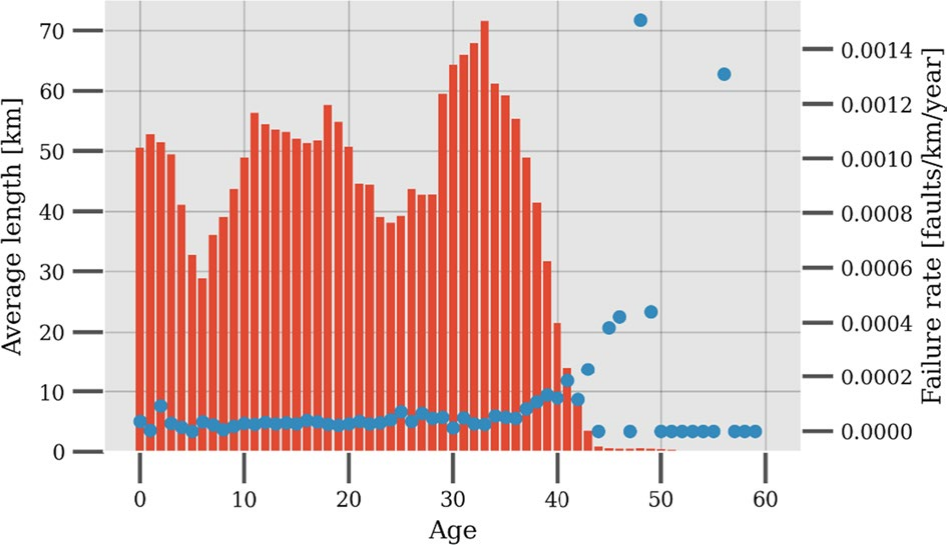


Figure 5 Histogram of the distribution of average length for each observation bin of 1 year. The blue dots denote the failure rate for each age bin

A population of pipes that are older than the observation period can be assumed to have developed faults at about the same rate, so the number of faults in those pipes in a given year can be calculated as the product of the failure rate of pipes at the age the pipes were at that time and the total length of the same cohort of pipes. This can be expressed as:

9 $${n}_{f}={\lambda }_{yb}{L}_{yb}$$

where ${n}_{f}$ is the number of faults, ${\lambda }_{yb}$ is the failure of pipes aged $yb$, and ${L}_{yb}$ is the total length of pipes aged $yb$. This assumption is used for all years in which the failure statistics for a population of same-age pipes was not recorded. Employing this imputation strategy results in 1075 additional faults scattered over the lifetime of the pipes. The feature vectors of all imputed fault observations are sampled randomly from the distribution of the observed pipes that have failed. This is an attempt to avoid wrongfully impacting the covariate coefficients of the WPHM.

Figure 6 shows the median survival probability based on the imputation of left-censored observations Compared to Figure 3, the survival probability declines faster, which means that expected life is lowered. This suggests that the imputation of left-censored observations can reduce the bias toward longer expected lives of survival models. Nevertheless, the expected life is still incredibly high, so the bias from left-censoring cannot alone explain the deviation between the model outcome and industry belief.
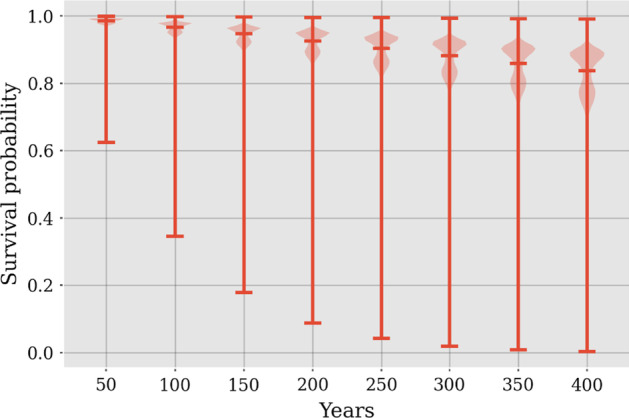


Figure 6 Violin plot of the median survival probability of the entire population of pipes at different service lives based on parameters determined based on the imputation of left-censored observations. The horizontal lines represent max (top), median (middle), and min (bottom) median survival probability

Another potential reason for the discrepancy is that the vast majority of pipes have likely not reached the wear-out stage of their lifecycle. The lifecycle is generally believed to be described by the bathtub curve, as presented in [22], which has three distinct stages. The first stage is described as infant mortality, which is caused by manufacturing or installation faults. The second stage is characterized by a constant failure rate, and the last stage, the wear-out stage, describes an increasing failure rate as the materials reach the end of their lifetime.

Firstly, infant mortality is not observed, see Figure 5. This is because these faults are detected using insulation resistance monitoring with copper wires integrated into the insulation and this datatype was not available for this study. The technology was adopted fairly recently, so it primarily pertains to the newest pipes. This is, of course, one reason why the number of observed faults is lower than the actual number of faults in this study and is likely to bias the model towards longer expected lives.

The failure rate is close to constant until the pipes reach an age of approximately 40 years. At this point, the amount of data used to calculate the failure rate is fairly low (low number of pipes and correspondingly low number of faults), meaning that the failure rates are very uncertain. This, coupled with the information that lifetime is expected to be 50 years at the very least, renders it challenging to confirm whether a small portion of the pipes has reached their wear-out stage. The time following the expected lifetime of the pipes is an important object of study, as it represents the wear-out stage’s increase in failure rate, and this object of study is unavailable in the dataset, as very few if any, pipes have reached the wear-out stage of their lifecycle.

Survival data for pipe networks are widely analysed in the water distribution domain. Old maintenance records are very helpful contributors in this context [10], as they can cover decades of maintenance data [3, 9, 10]. However, several studies emphasize that an extensive maintenance database is not necessary for accurate survival modeling [4, 2, 23], e.g. [4] states that “*short maintenance records (5–10 years) give as good results as long maintenance records*”. Additionally, multiple studies suggest that survival analysis can be useful even under left-censoring or left-truncation [4, 23]. However, left-censoring and left-truncation can still create bias in survival modeling by underestimating the apparent time to failure, which we show is not the only bias in the current model.

The general characteristic of the water distribution domain’s survival data is that at least parts of the pipe network are older than their expected life [3, 9, 10]. A concrete example of this is the network in [2], which is more than 50 years old while the expected lifetime of the network’s pipes is approximately 30 years. This means that the evolution of the pipes’ failure rates is well expressed in the maintenance and asset data. Performing survival analysis on relatively young pipe networks, therefore, runs the risk of not having the pipes’ wear-out phase represented in the data.

While this can explain a model bias towards much longer estimates of the service life of district heating pipes, it is also possible that the district heating network is much more reliable than initially thought. It is possible that the Danish district heating association have underestimated the expected life of the district heating grid. However, the order of magnitude difference between the expectations and the model output makes it very unlikely that this is the only explanation.

CONCLUSION

This paper explored probabilistic survival modeling for pipes used in district heating and found that the model predicted a much longer service life for the pipes than the industry expects. We identified several reasons for this discrepancy, with truncation and censoring bias being two of them. This bias can be reduced by imputating censored observations—however, even with imputation, the model was still biased towards much longer lifetimes than expected. A short review of survival analysis in the water distribution domain suggested that the primary reason for the large discrepancy is that the case network is relatively young, so the increase in failure rate during the wear-out stage of the pipes’ lifecycle is not well represented in the data. Good data collection for survival analysis, based on the work presented in this paper, entails tracking all failures from when the pipe network is commissioned, though imputation can help if this is not done. Lastly, survival analysis is more applicable for relatively old infrastructures for which the data describes all parts of the pipes’ lifecycle.

Future research might perform survival analysis using more complete data, with less need to censor observations, which represent an older network. This would provide a case study, where the findings of this paper could be corroborated. Additionally, the value of censor-adjusted survival modeling could be studied and demonstrated.

**List of abbreviations**

- PHM: Proportional Hazard Models
- WPHM: Weibull proportional hazard model
- GIS: geographic information system

**Acknowledgements:** This work was supported by two projects: “IEA DHC TS4” no. 134-22011 and “Proactive and Predictive Maintenance of District Heating Systems” no. 64020-2102 by the funding agency Danish Energy Agency under the Energy Technology and Development and Demonstration Programm, Denmark.

**References**

1. S. Yamijala, S. D. Guikema and K. Brumbelow, “Statistical models for the analysis of water distribution system pipe break data,” *Reliability engineering and system safety,* vol. 94, pp. 282–293, 2009.
2. S. E. Christodoulou, “Water network assessment and reliability analysis by use of survival analysis,” *Water resource management,* vol. 25, pp. 1229–1238, 2011.
3. G. Kabir, S. Tesfamariam and R. Sadiq, “Predicting water main failures using bayesian model averaging and survival modelling approach,” *Reliability engineering and system safety,* vol. 142, pp. 498–514, 2015.
4. Y. Le Gat and P. Eisenbeis, “Using maintenance records to forecast failures in water networks,” *Urban water,* vol. 2, no. 3, pp. 173–181, 2000.
5. S. Park, H. Jun, N. Agbenowosi, B. J. Kim and K. Lim, “The proportional hazards modeling of water main failure data incorporating the time-dependent effects of covariates,” *Water resource management,* vol. 25, pp. 1–19, 2011.
6. S. Rimkevicius, A. Kaliatka, M. Valincius, G. Dundulis, R. Janulionis, A. Grybenas and I. Zutautaite, “Development of approach for reliability assessment of pipeline network systems,” *Applied Energy,* no. 94, pp. 22–33, 2012.
7. I. Postnikov and V. Stennikov, “Modifications of probabilistic models of states evolution for reliability analysis of district heating systems,” in *The 6th International Conference on Power and Energy Systems Engineering*, Okinawa, 2019.
8. M. Valincius, I. Zutautaite, G. Dundulis, S. Rimkevicius, R. Janulionis and R. Bakas, “Integrated assessment of failure probability of the district heating network,” *Reliability Engineering and System Safety,* no. 133, pp. 314–322, 2015.
9. B. Rajani, Y. Kleiner and J.-E. Sink, “Exploration of the relationship between water main breaks and temperature covariates,” *Urban water journal,* vol. 9, no. 2, pp. 67–84, 2012.
10. D. Fuchs-Hanusch, B. Kornberger, F. Friedl, R. Scheucher and H. Kainz, “Whole of life cost calculations for water supply pipes,” *Water asset management international,* vol. 8, no. 2, pp. 19–24, 2012.
11. B. Snider and E. McBean, “Combining machine learning and survival statistics to predict remaining service life of watermains,” *Journal of infrastructure systems,* vol. 27, no. 3, 2021.
12. L. K. Mortensen, H. R. Shaker and C. T. Veje, “Relative fault vulnerability prediction for Energy Distribution Networks,” *Applied Energy,* vol. 322, p. 119449, 2022.
13. A. Scheidegger, J. P. Letião and L. Scholten, “Statistical failure models for water distribution pipes a review from a unified perspective,” *Water Research,* vol. 83, pp. 237–247, 2015.
14. S. Kotz and N. L. Johnson, “Introduction to Cox (1972) regression models and life-tables,” in *Breakthroughs in statistics volume 2*, New York, Springer, 1992, pp. 519–526.
15. J. Albert, Bayesian computation with R, New York: Springer, 2009.
16. J. A. Turkson, F. Ayiah-Mensah and V. Nimoh, “Handling censoring and censored data in survival analysis: A standalone systematic literature review,” *International journal of mathematics and mathematical sciences,* vol. 2021, 2021.
17. S. G. Walker, “Sampling unnormalized probabilisties: An alternative to the Metropolis–Hastings algorithm,” *SIAM Journal on Scientific Computing,* vol. 36, no. 2, pp. 482–494, 2014.
18. OpenStreetMap, “Denmark, shape file,” Online. Available: https://download.geofabrik.de/europe.html. Accessed 3 9 2021.
19. GEUS, “Jordartskort 1:200 000, shape-filer, shape file,” Online. Available: https://frisbee.geus.dk/geuswebshop/. Accessed 3 9 2021.
20. GEUS, “Kort over dybden til redoxgrænsen, pdf og shapeformat, shape file,” Online. Available: https://frisbee.geus.dk/geuswebshop/. Accessed 3 9 2021.
21. Dansk Fjernvarme, “Levetid for ledninger,” 31 January 2022. Online. Available: https://www.danskfjernvarme.dk/viden-og-v%C3%A6rkt%C3%B8jer/ledningsnet-og-lagring/levetid-for-ledninger. Accessed 12 April 2022.
22. K. Sernhed and M. Jönson, “Risk management for maintenance of district heating networks,” *Energy Procedia,* vol. 116, pp. 381–393, 2017.
23. S. Alvisi and M. Franchini, “Comparative analysis of two probabilistic pipe breakage models applied to a real water distribution system,” *Civil engineering and environmental systems,* vol. 27, no. 1, pp. 1–22, 2010.
24. Z. Zhang and J. Sun, “Interval censoring,” *Statistical methods in medical research,* vol. 19, no. 1, pp. 53–70, 2010.

## Design of data management service platform for intelligent electric vehicle charging controller—multi-charger model

### Pedro Baptista^1^, Jose Rosado^2,4^, Filipe Caldeira^1,3^, Filipe Cardoso^1,4^

#### ^1^Viseu Polytechnic - ESTGV, Viseu, Portugal; ^2^Coimbra Polytechnic - ISEC, Coimbra, Portugal; ^3^CISeD IPV, Viseu, Portugal; ^4^INESC Coimbra, Coimbra, Portugal

##### **Correspondence:** Filipe Cardoso (fcardoso@estgv.ipv.pt)

*Energy Informatics* 2022, 5(3)

**Summary:** The electric charging solutions for the residential market imply, in many situations, an increase in the contracted power in order to allow to perform an efficient charging cycle that starts when the charger is connected and ends when the VE battery is fully charged. However, the increase in contracted power is not always the best solution for faster and more efficient charging. With a focus on the residential market, the presented architecture is suitable for single-use and shared connection points, which are becoming common in apartment buildings without a closed garage, allowing for sharing the available electrical connections to the grid. The multi-charger architecture allows using one or several common charging points by applying a mesh network of intelligent chargers orchestrated by a residential gateway. Managing the generated data load involves enabling data flow between several independent data producers and consumers. The data stream ingestion system must be scalable, resilient, and extendable.

**Keywords:** EVSE, Electric Vehicles, Intelligent Charging, Load Management, Mobility, Mesh, Data Management, Fog Computing

INTRODUCTION

Electric Vehicle (EV) are environmentally friendly since they do not emit any gas directly into the atmosphere, have fewer maintenance needs and operating expenses, and offer a quieter driving experience [1, 2]. These are the primary advantages of EV, which are becoming more and more attractive as the technology evolves. Even though they presently represent only 2.7% of global sales, according to the Bloomberg report by [3], the tendency is for them to grow. It is predicted that by 2025, EV will account for 10% of worldwide passenger vehicle sales, growing to 28% in 2030 and 58% in 2040, respectively. According to an analysis conducted by the Association of Electric Vehicles User (UVE) for Portugal, the sale of EV increased by 80% in November 2020 when compared to the same month in 2019 [4].

In many situations, the EV charging solutions for the home market implies an increase in the contracted power to allow for an efficient charging cycle that begins when the charger is connected and stops when the EV battery’s maximum charge is reached. Increased contracted power is not necessarily the most effective approach for charging faster and more efficiently. A limited power grid connection shared among a large number of tenants makes it difficult to implement electric charging solutions able to solve challenges such as, controlling expenses by user, optimizing charging time, and even balancing the load based on the energy available at a given time [5, 6].

The authors in [7] present two distinct Intelligent Electric Vehicle Charging Controller (IEVCC) system configurations. This work focuses on the Mesh version intended for use in condominiums. In this scenario, tenants do not have access to parking spots with independent electrical connections, and the only solution available is to share the building’s common grid. With the difficulties identified when designing and implementing multi-client solutions in mind, this work proposes a technical architecture capable of managing high data loads. The solution must be resilient and scalable to address the mesh installation problems and optimize grid usage. These aspects will benefit the end consumers and also assist the electricity distributors.

Multiple architectures were described using protocols like MQTT or Zigbee [8, 9]. The common gap is the detailed description of the software stack and how each layer interrelates. Some more detailed articles [10], in what relates to the software stack don’t approach high data load scenarios where the scalability and flexibility of the solution is critical.

The planned solution is considered a streaming analytics system, typically consisting of three layers: ingestion, processing, and storage. The ingestion layer is the gateway to streaming. Data flow from inputs to processing and storage levels is decoupled, automated and managed. The processing layer receives the ingestion layer’s data streams and transfers the output or intermediate results to storage. The storage layer keeps data in memory for iterative calculations or in databases for long-term storage. The analytics findings are given to a range of display and decision-assistance tools [11, 12].

This paper is organized as follows: after this introduction, the different data stages are presented in Section II. In Section III the full architecture diagram is presented and discussed, finally, the Conclusions are presented in Section IV.

CHARGING DATA STAGES

According to [13], on the Internet of Things, there are five primary data processing architectures, fog-based processing, middleware-based processing, cloud-based processing, cloudlet computing, and mobile-edge computing.

The current data load comes from chargers, electrical counters, and message handling devices (broker). While systems operate, it is vital to log and store not only charging data, but also device status and usage metrics. To minimise damage in the event of network or device failures, the local setup (device layer) must be able to store and recover from failures. The final architecture proposal will take that into consideration.

The next natural step is to store data in the cloud, but once again, due to the sensitive nature of data and the significant load caused by each local instance, transmitting gathered data straight to cloud servers has proven to be challenging [14]. The same article discusses how fog computing may help minimize cloud reliance while improving performance. Nevertheless, the paper concludes that cloud and fog are complementary and can help deliver better and more complete services.

Fog Architecture

Cloud computing and fog computing infrastructures do not compete with each other, and they’re complementary architectural solutions. IoT applications connect across fog nodes, and devices must be linked to at least one of these fog nodes (Fig. 1). Any device part of the IEVCC solution may connect to fog nodes which may be used in specific geographical cloud areas [15]. Because each fog node is a single point of failure, its spread and replication across regions should be considered for failure recovery and redundancy.

Multi-Charger Model

The multi-charger installation includes multiple chargers and may also include multiple electrical counters. Figure 2 illustrates how each device connects to the “heart” of the device layer (Device Manager). This device is responsible for message handling and forwarding. At the same time, it manages authorized devices and clients during charges.

Each instance can be configured with custom load balancing rules, charger priority, and energy source selection when more than one source is available or when the provider shares the current source through an Application Programming Interface (API) endpoint. All data is stored locally and forwarded to remote instances for data cleansing and transformation. Electrical communication usage, system logs, and client usage are then available for access by clients and providers.

DESIGN OF DATA MANAGEMENT SERVICE PLATFORM

In order to allow data flow between several independent data producers and consumers, a data stream ingestion system must be scalable, resilient, and extendable. Chargers and electrical counters are the primary focus of the current configuration. However, the Device Manager design ensures that more Internet of Things devices will be able to connect and integrate into the solution in the future.
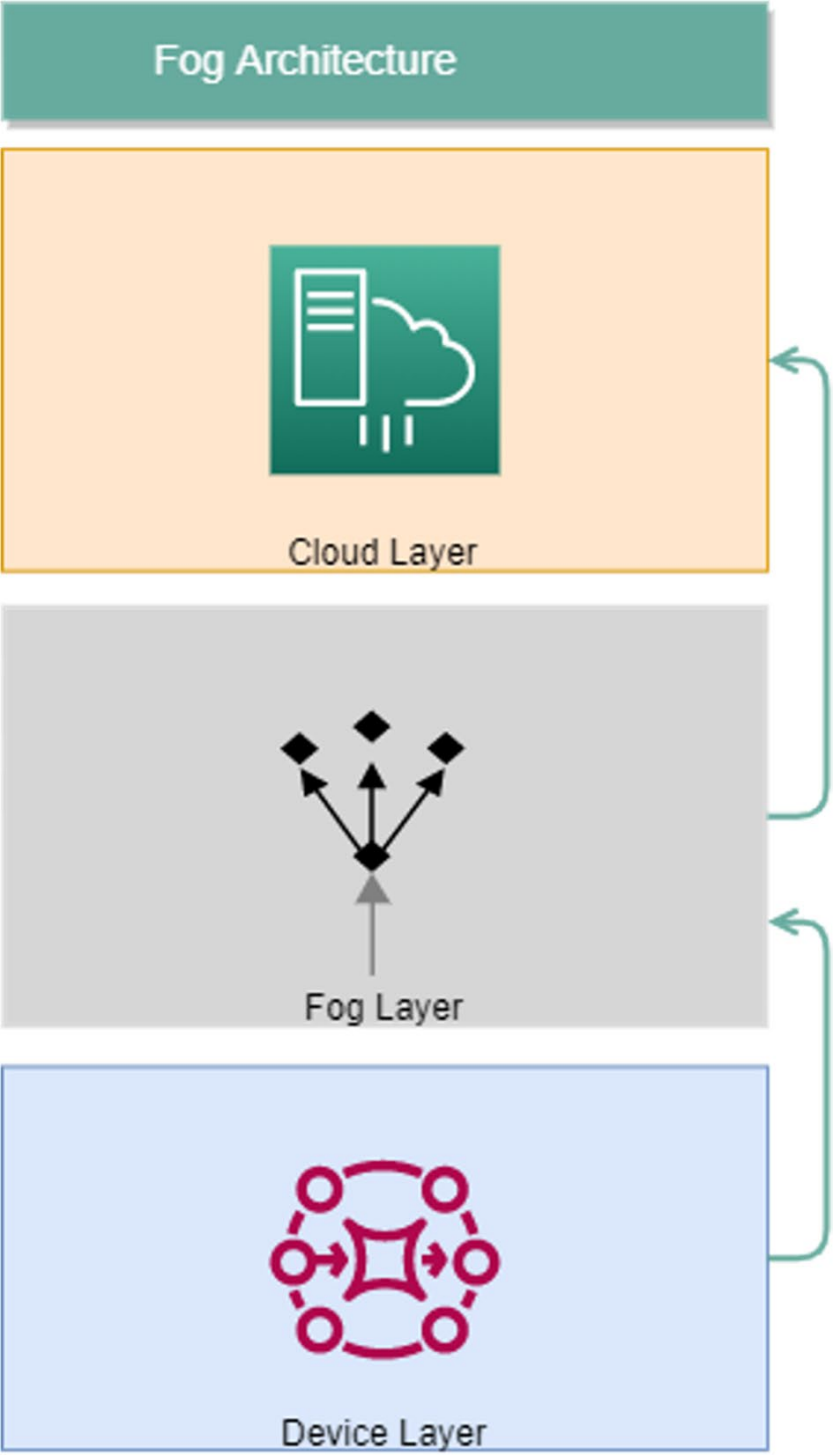


Figure 1 Fog architecture

In order to demonstrate how to integrate the Fog Architecture into the Multi-Charger Model, the Fig. 3 depicts the position of each entity within the three Fog Architecture levels. Each colored section maps the three distinct layers (Device, Fog and Cloud layers), where generated data is saved and then forwarded to the subsequent layer instances. It is crucial to clean and aggregate each record to be stored in the database during this process. It is also important to note that while generated data flow in one direction only, it is mandatory to authenticate users, devices, charging sessions, and others. This responsibility is taken care of by the Device Manager through the API instance in the cloud layer.

Device Layer

The device layer includes all the devices that support the local area network, like routers, switches, wireless access points or extenders, and all the smart devices connected locally. The smart ESP32-based devices are chargers and electricity meters for the current solution. Multiple other IoT devices may be integrated into the solution in the future.

The solution’s heart is the Device Manager. Raspberry Pi version 3 boards were tested during development with no performance issues while handling device auth, messages, local storage, and data forwarding to the fog layer. For the Manager role, it is clear that power consumption and price will affect the board choice. Given that architecture compatibility is not an issue, the minimum requirements must meet the Raspberry Pi 3 specifications, as well as the ability to run Docker.
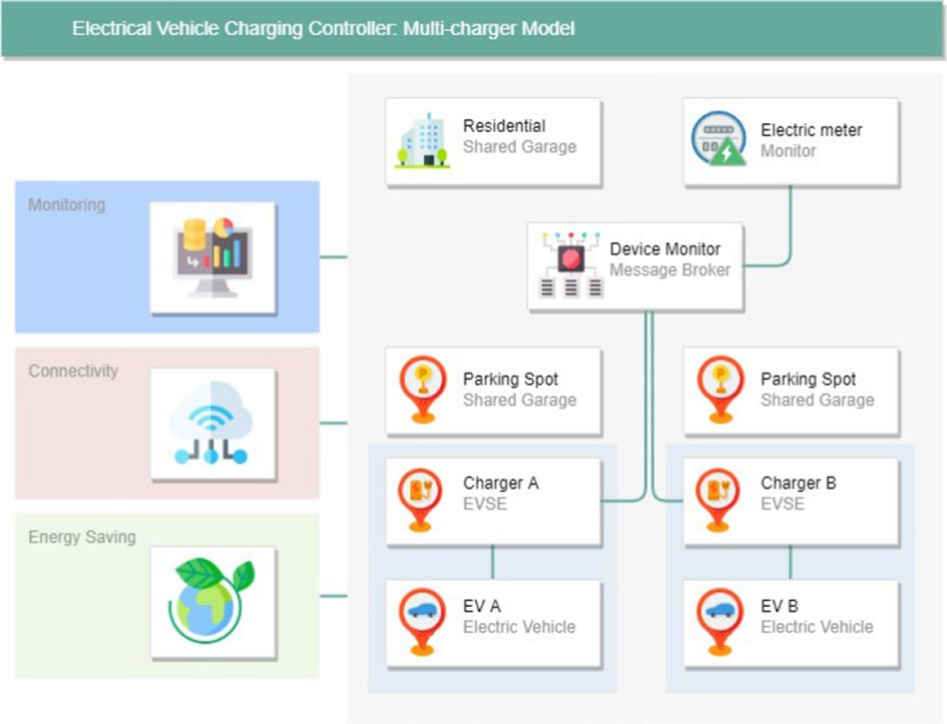


Figure 2 Multi-charger model

Fog Layer

The Fog Layer comprises devices in an intermediate layer between the cloud and the Device Layer. In this case, data is transferred to and processed by a computer or data center regionally located. Splitting this processing power across multiple regions decreases the total load each fog node will handle while increasing redundancy, a significant concern when dealing with critical data.

Fog node hardware must meet the minimum system requirements set for the distributed event streaming platform and the applications for cleansing and transforming data. Our prediction suggests each node has 8 GB of RAM, 4 CPU cores, 1 TB of storage, and 1GbE connection.

Cloud Layer

The cloud computing infrastructure builds on top of large-scale clusters that run various applications and pursue the core foundation that enables computing resources to be used to their full potential. Cloud customers expect the entire system to be reliable, with redundant network and hardware. These cloud solutions allow companies to access data storage, resources, and on-demand services over the internet. Although cloud providers offer a variety of solutions for several operations, based on the presented Mesh-Model (Fig. 3), the core business activities in the Cloud Layer include database services and API web applications. The Cloud Layer is sub-layered into 3 layers: Infrastructure as an Infrastructure as a Service (IaaS), Platform as a Service (PaaS), and Software as a Software as a Service (SaaS). Choosing the best cloud layer depends on the budget, resources, the size of the operations, and multiple other factors.
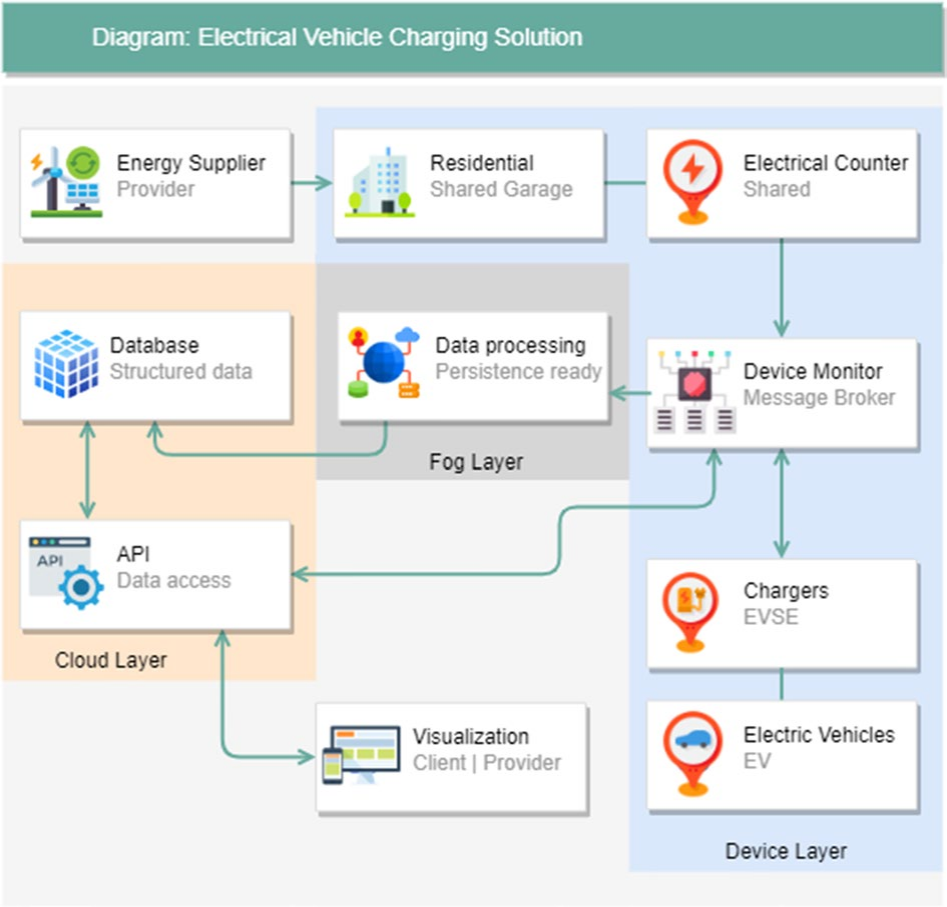


Figure 3 Mesh-model diagram

Tech Stack

A tech stack is a company’s choice of technologies to develop and manage an app or project. A tech stack often includes programming languages, frameworks, databases, front-end and back-end tools, and apps linked through API [16].

In a top-to-bottom analysis of Fig. 4, in the Device Layer, the current charger and electricity meter devices are programmed in C++, while the Device Manager is currently being developed in Python 3.8, with a tested compatible version range from Python 3.6 to Python 3.10. MQTT message broker (Mosquitto MQTT) and InfluxDB, an open source Time Series Database, both run on the same hardware. Each fog node in the Fog Layer will provide one or more Apache Kafka instances, an open-source distributed event streaming platform. Apache Kafka advertises necessary core capabilities like high throughput with low latency (2 ms), being prepared to scale, and delivering high availability. It’s also important to mention the built-in stream processing that enables the processing of event streams using joins, aggregations, filters, transformations, and exactly-once processing. It is also worth mentioning that the Kafka Connect interface is pre-integrated with hundreds of event sources and sinks, including Postgres, Java Message Service (JMS), Elastic-search, and Amazon Web Services (AWS) S3.

As previously stated, in the Cloud Layer, our solution’s core depends on a Post-greSQL database instance and the possibility to host web applications like client portals or API to access and store information. The best cloud solution for PostgreSQL service is still open for further analysis, howsoever it is mandatory to have scalability possibilities, backups, and snapshots. Multi-region availability and synchronization will be decisive when dealing with thousands of clients. Region and response time are essential factors in web application hosting, but so are high availability with load balancing, security, and scalability.
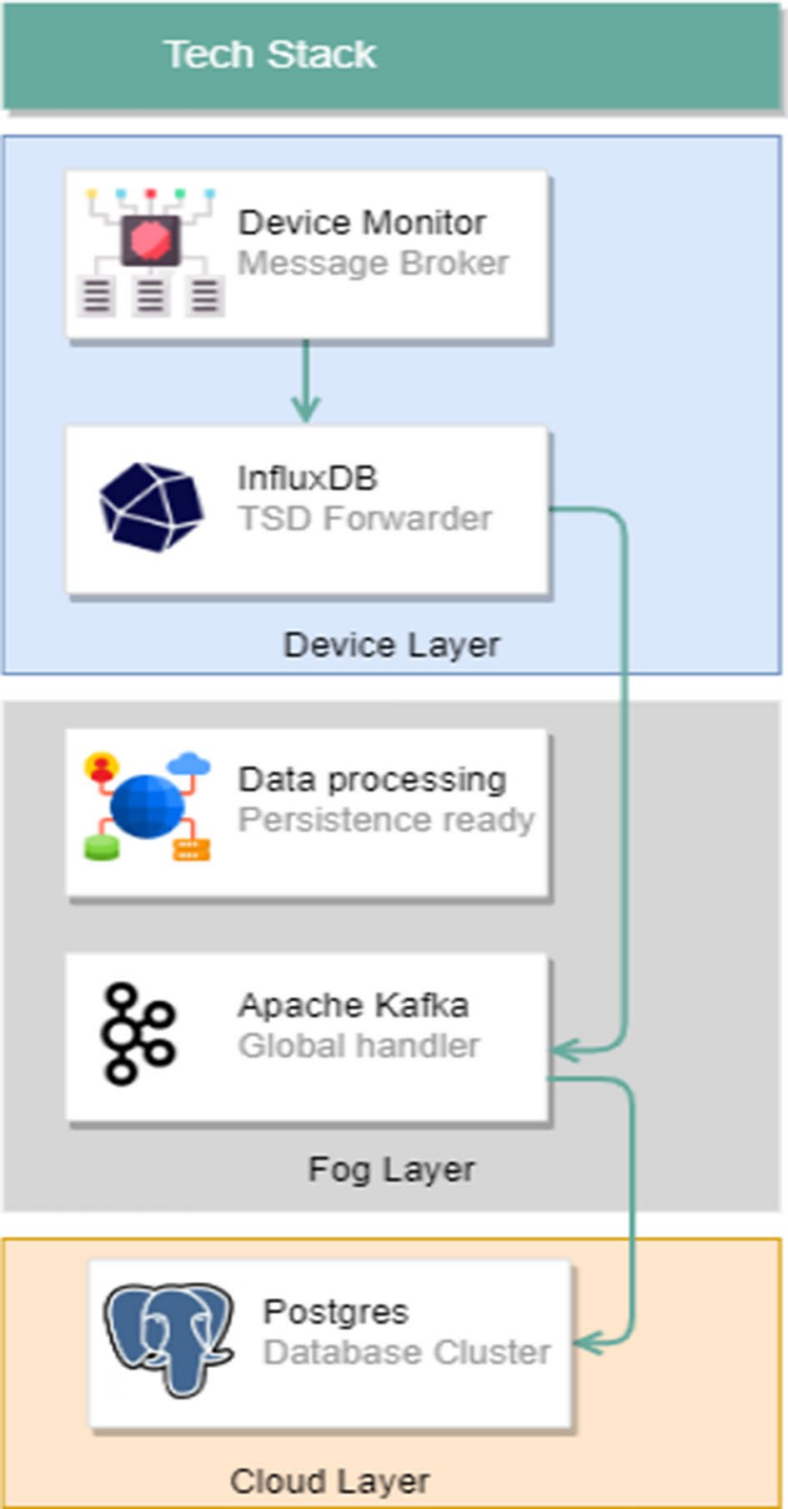


Figure 4 Tech stack

DISCUSSION

During the investigation process, we analyzed multiple designs for software solutions with similar requirements. Despite the simplicity of adopting one component for the software stack instead of another, work must be done while trying to describe how each layer interrelates. The specification of infrastructure needs for a large data load is a difficult undertaking that involves numerous factors and criteria, such as data types, scalability and type of processing, communication between tasks or processes, and so on. Software engineering is crucial in ensuring that such workloads make the most use of the underlying hardware resources. As an example, data created by the current setup is handled by a cloud solution setup designed to leverage dynamic and adaptive cluster resource management, dimensioning, and configuration based on economic cost, quality of service, and availability requirements. Agile frameworks are typically used in this application design to lower barriers between development and operations teams, accelerate workflows (i.e. high deployment rates for faster feedback, better code quality leading to fewer errors and lower costs, and so on), and increase the reliability, stability, and resilience of the production environment.

CONCLUSIONS

Ingestion of data is critical for businesses and organizations that gather and analyze massive amounts of data. Continuous data streams are often ingested into big data processing and management systems from external sources. They are either processed incrementally or used to create a persistent dataset and related indexes. In order to keep up with vast amounts of rapidly changing data, stream processing systems must be able to ingest, analyze, and persist data continuously.

This work presents an architecture and the corresponding tech stack designed to handle massive time-critical data while performing cleansing and transforming operations, then storing it in a cloud database service. The innumerable options for each entity in the tech stack open new paths to different approaches and bench-marks. This will also help choose the best-tailored cloud provider for the solution’s specific needs.

**Acronyms**

- API: Application Programming Interface
- AWS: Amazon Web Services
- EV: Electric Vehicle
- IaaS: Infrastructure as a Service
- IEVCC: Intelligent Electric Vehicle Charging Controller
- IoT: Internet of Things
- JMS: Java Message Service
- MQTT: MQ Telemetry Transport
- PaaS: Platform as a Service
- SaaS: Software as a Service
- UVE: Association of Electric Vehicles User

**Acknowledgements:** This work is partially funded by National Funds through the FCT—Foundation for Science and Technology, I.P., within the scope of the projects UIDB/00308/2020, UIDB/05583/2020. Furthermore, we would like to thank the Research Centre in Digital Services (CISeD), the Polytechnics of Viseu, Polytechnics of Coimbra and INESC Coimbra for their support.

**References**

1. Dubey A, Santoso S. Electric Vehicle Charging on Residential Distribution Systems: Impacts and Mitigations. IEEE Access. 2015;3:1871–1893.
2. Aghabali I, Bauman J, Kollmeyer PJ, Wang Y, Bilgin B, Emadi A. 800-V Electric Vehicle Powertrains: Review and Analysis of Benefits, Challenges, and Future Trends. IEEE Transactions on Transportation Electrification. 2021;7(3):927–948.
3. Henbest S, Kimmel H, Callens J, Vasdev A, Brandily T, Berryman I, et al. New Energy Outlook 2021: BloombergNEF: Bloomberg Finance LP. BloombergNEF; 2020. Accessed on 15.06.2022. Available from: https://about.bnef.com/new-energy-outlook/.
4. Nascimento M. Vendas de Ve´ıculos El´etricos em novembro de 2020 aumentam 80% em rela¸c˜ao a novembro de 2019; 2020. Accessed on 12.06.2022. Available from: https://www.uve.pt/page/vendas-ve-11-2020/.
5. Shepelev A, Chung CY, Chu CC, Gadh R. Mesh network design for smart charging infrastructure and electric vehicle remote monitoring. In: 2013 International Conference on ICT Convergence (ICTC); 2013. p. 250–255.
6. Ayan O, Turkay B. Domestic electrical load management in smart grids and classification of residential loads. In: 2018 5th International Conference on Electrical and Electronic Engineering (ICEEE); 2018. p. 279–283.
7. Cardoso F, Rosado J, Silva M, Teixeira CJC, Agreira CIF, Caldeira F, et al. Intelligent Electric Vehicle Charging Controller. In: 2021 IEEE Vehicle Power and Propulsion Conference (VPPC); 2021. p. 1–5.
8. Lee ZJ, Chang D, Jin C, Lee GS, Lee R, Lee T, et al. Large-Scale Adaptive Electric Vehicle Charging. In: 2018 IEEE International Conference on Communications, Control, and Computing Technologies for Smart Grids (SmartGridComm); 2018. p. 1–7.
9. Qian K, Brehm R, Ebel T, Adam RC. Electric Vehicle Load Management: An Architecture for Heterogeneous Nodes. IEEE Access. 2022;10:59748–59758.
10. Komasilovs V, Zacepins A, Kviesis A, Marinescu C, Serban I. Development of the Web Platform for Management of Smart Charging Stations for Electric Vehicles. In: VEHITS; 2018. p. 595–599.
11. Isah H, Zulkernine F. A Scalable and Robust Framework for Data Stream Ingestion. In: 2018 IEEE International Conference on Big Data (Big Data); 2018. p. 2900–2905.
12. Dias de Assunção M, da Silva Veith A, Buyya R. Distributed data stream processing and edge computing: A survey on resource elasticity and future directions. Journal of Network and Computer Applications. 2018;103:1–17. Accessed on 15.06.2022. Available from: https://www.sciencedirect.com/science/article/ pii/S1084804517303971.
13. Aung TT, Thaw AM, Zhukova NA, Man T, Chernokulsky VV. Data processing model for mobile IoT systems. Procedia Computer Science. 2021;186:235–241. 14th International Symposium”Intelligent Systems—Accessed on 14.06.2022. Available from: https://www.sciencedirect.com/science/article/pii/S1877050921009583.
14. Garcia J, Simó E, Masip-Bruin X, Marín-Tordera E, Sànchez-López S. Do We Really Need Cloud? Estimating the Fog Computing Capacities in the City of Barcelona. In: 2018 IEEE/ACM International Conference on Utility and Cloud Computing Companion (UCC Companion); 2018. p. 290–295.
15. Kanyilmaz A, Cetin A. Fog Based Architecture Design for IoT with Private Nodes: A Smart Home Application. In: 2019 7th International Istanbul Smart Grids and Cities Congress and Fair (ICSG); 2019. p. 194–198.
16. Limón AT, Schulaka C. What’s in Your Tech Stack? Journal of Financial Planning. 2020;33(2):22–27.

